# Computational and experimental approaches to chart the *Escherichia coli* cell-envelope-associated proteome and interactome

**DOI:** 10.1111/j.1574-6976.2008.00141.x

**Published:** 2008-12-01

**Authors:** Juan Javier Díaz-Mejía, Mohan Babu, Andrew Emili

**Affiliations:** Banting and Best Department of Medical Research, Terrence Donnelly Center for Cellular and Biomolecular Research, University of TorontoToronto, ON, Canada

**Keywords:** cell-envelope, *Escherichia coli*, subcellular localization, algorithms, bioinformatics, proteomic methods

## Abstract

The bacterial cell-envelope consists of a complex arrangement of lipids, proteins and carbohydrates that serves as the interface between a microorganism and its environment or, with pathogens, a human host. *Escherichia coli* has long been investigated as a leading model system to elucidate the fundamental mechanisms underlying microbial cell-envelope biology. This includes extensive descriptions of the molecular identities, biochemical activities and evolutionary trajectories of integral transmembrane proteins, many of which play critical roles in infectious disease and antibiotic resistance. Strikingly, however, only half of the *c*. 1200 putative cell-envelope-related proteins of *E. coli* currently have experimentally attributed functions, indicating an opportunity for discovery. In this review, we summarize the state of the art of computational and proteomic approaches for determining the components of the *E. coli* cell-envelope proteome, as well as exploring the physical and functional interactions that underlie its biogenesis and functionality. We also provide a comprehensive comparative benchmarking analysis on the performance of different bioinformatic and proteomic methods commonly used to determine the subcellular localization of bacterial proteins.

## Introduction

The cell-envelope of Gram-negative bacteria, for example *Escherichia coli*, can be defined as an organelle composed by: (i) a phospholipidic inner membrane (IM), also called the cytoplasmic membrane, (ii) the periplasm, which is a gel-like structure intimately related with the cell wall, consisting of a structurally rigid peptidoglycan layer, and (iii) an outer membrane (OM) formed by phospholipids and lipopolysaccharide. In contrast, Gram-positive bacteria such as *Bacillus subtilis* possess a cytoplasmic membrane along with a thicker cell wall, and lack an OM. The cell-envelope plays an important role for pathogenic bacteria during host invasion, colonization and evasion of the immune system and so is a major target of current antimicrobials. Common antibiotics such as the β-lactams (e.g. penicillin, amoxicillin) perturb the synthesis and/or the stability of the cell-envelope, specifically disrupting the cell-wall biogenesis, leading to loss of selective permeability and osmotic integrity, resulting in bacterial cell death.

According to bioinformatic predictions, the set of proteins putatively spanning the membranes constitute *c*. 25–30% of the entire proteome in species from the three domains of life (Wallin & von Heijne, 1998). In the case of *E. coli*, these include *c*. 900 transmembrane proteins spanning the IM (hereafter called TIMPs) and *c*. 90 spanning the OM (hereafter called TOMPs) (see The *E. coli* cell-envelope compartments and their associated proteomes section). Likewise, the periplasmic proteins make important contributions to membrane biology. In *E. coli c*. 250 proteins, representing *c*. 6% of all predicted water-soluble proteins, are located in the periplasm (Gardy *et al.*, 2005). Almost as a rule, each membrane is spanned by a specific type of protein secondary structure element: the TIMPs span the IM via α-helices, while the TOMPs span the OM via β-barrels, with the notable exception of Wza, a protein involved in the export of capsular polysaccharides whose recently determined three-dimensional (3D) structure surprisingly revealed α-helices spanning the OM (Dong *et al.*, 2006).

**Fig. 1:**
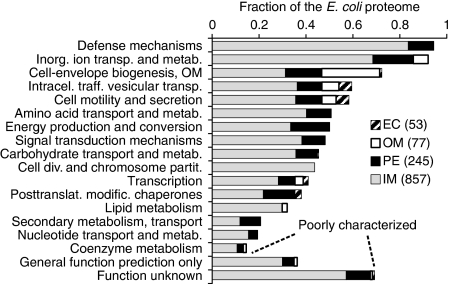
A general functional classification of the *Escherichia coli* cell-envelope related proteome. A set of 1179 proteins tentatively forming the cell-envelope proteome of *E. coli* K-12 (substrain W3110) was selected combining the results of four different predictors of protein global subcellular localization by ‘Majority Consensus’ (see section ‘Majority Consensus’ improves the prediction of global subcellular localization for details). The number of proteins for each compartment forming the ‘Majority Consensus’ is shown in parentheses. Fractions represent the number of proteins in each functional category – according to the COGs database (Tatusov *et al.*, 2000) – divided by the total number of *E. coli* proteins in the respective category. In comparison with the cytoplasmic proteins (the remaining fraction not shown in each functional category), the cell-envelope proteome is markedly enriched in proteins with an unknown function (*c*. 70%). Two COG categories, namely Translation and DNA replication, recombination and repair, are not shown, as none of these 1179 proteins is classified into such categories. IM, inner membrane; PE, periplasmic; OM, outer membrane; EC, extracellular.

A systematic survey of protein functional classification databases like Clusters of Orthologous Groups of proteins (COGs) (Tatusov *et al.*, 2000) indicates that virtually the entire spectrum of core biological functions is present in the cell-envelope-related proteome, with the exception of factors directly involved in DNA replication and certain cytoplasmic metabolic branches (Fig. 1). For example, the *E. coli* IM hosts over 250 transporters for sugars, amino acids, etc., as well as *c*. 30% (72/239) of all proteins involved in energy production and *c*. 90% (30/33) of proteins implicated in defense. In turn, the periplasm is enriched with *c*. 20 proteins aiding the folding and mobilization of other proteins from the cytoplasm through the cell-envelope. Additionally, the OM hosts *c*. 15 highly abundant porin channels that act as permeability determinants, protecting the cell from potentially harmful compounds in its environment (e.g. antibiotics).

Yet although at least 60 proteins associated with the cell-envelope are encoded by essential genes (Baba *et al.*, 2006) and hence are potential targets for antimicrobials, currently only fewer than half of the *c*. 30 bacterial proteins targeted by prescription drugs are associated with the cell-envelope (Haselbeck *et al.*, 2002). Moreover, about *c*. 500 putative TIMPs, TOMPs and periplasmic proteins remain functionally uncharacterized (Fig. 1). Given the broad biological and clinical significance of the bacterial cell-envelope, acquiring a more complete understanding of its components and their associations should suggest rational new targets for antibiotic development.

Because genes and proteins do not act in isolation, one of the main challenges for ‘Systems Biology’ is to understand how cellular processes are functionally integrated at the molecular level. This requires a global perspective on the various types of interactions (i.e. physical, metabolic, regulatory, epistatic, etc.) that occur between gene products, which in turn are organized into multimeric protein complexes, pathways and functional modules. Nonetheless, determining the proteome and dynamic interactions occurring in the cell-envelope itself represents a significant challenge for both experimentalists and bioinformaticians alike. For example, transmembrane proteins possess hydrophobic regions that make them difficult to solubilize and purify using conventional proteomic techniques, necessitating the application of specialized methods. In the computational biology domain, comparative genomic analyses of transmembrane proteins must be managed with caution because transmembrane regions often possess highly repetitive sequences that are commonly ignored (masked) by conventional sequence comparison tools and that therefore require specialized substitution matrices for proper sequence alignments.

In this review, we provide a summary of proteomic and bioinformatic approaches devoted to decipher the bacterial cell-envelope-related proteome and the myriad of physical interactions among its many components, using *E. coli* as a reference model. Our goal is not to provide a detailed description of such techniques, because several excellent in-depth reviews have been recently published for both proteomic (Krause, 2006; Hooker *et al.*, 2007; Weiner & Li, 2008; Poetsch & Wolters, 2008) and bioinformatic (Gardy & Brinkman, 2006; Punta *et al.*, 2007) approaches. Instead, we highlight some key benefits, and caveats, associated with the use of such tools, providing illustrative comparative studies where possible.

This review is divided into two major sections: the first addresses tools used for elucidating the putative cell-envelope proteome, which can be represented as nodes within a molecular interaction network, and the second is focused on experimental methods to examine the physical and functional interactions between such nodes, in particular, the detection of protein–protein interactions (PPIs) and functional relatedness using recently developed high-throughput phenotypic assays. Additionally, we provide a compilation of the *c*. 1200 proteins forming the *E. coli* K-12 cell-envelope-predicted proteome according to different proteomic and bioinformatic tools and their current annotations in various databases, together with an update of previous studies (Rey *et al.*, 2005b; Gardy & Brinkman, 2006) assessing the performance of these tools.

## *Escherichia coli* as a model

*Escherichia coli*, the historical workhorse of bacterial genetics and biochemistry, is ideally suitable for large-scale investigations of bacterial gene and protein function. To date, the fully sequenced genomes of three *E. coli* K-12 reference laboratory substrains (MG1655, W3110 and DH10B) are publicly available (Pruitt *et al.*, 2005), as well as three other nonpathogenic and six pathogenic isolates (Spears *et al.*, 2006). The Keio deletion strain collection (Baba *et al.*, 2006) provides single-gene knock-outs of 3985 *E. coli* K-12 nonessential genes (at least under standard laboratory growth conditions) and can be used, for example, in the systematic determination of gene or protein function based on systematic genome-wide phenotypic assays (Butland *et al.*, 2008; Typas *et al.*, 2008). Additionally, specialized metabolic databases such as EcoCyc (Keseler *et al.*, 2005) and gene transcription regulation resources such as RegulonDB (Gama-Castro *et al.*, 2008) cumulatively provide some degree of functional annotation for most (3511 out of the *c*. 4200; or *c*. 83%) of all the *E. coli* K-12 genes. Similarly, GenProtEC (Serres *et al.*, 2004) provides a hierarchical functional classification for *c*. 87% of *E. coli* K-12 genes, including 2583 (*c*. 61%) with experimentally supported gene annotations and 1097 (26%) with bioinformatic predictions, while the remaining 13% lack even tentative associated functions. A compilation of current *E. coli* protein annotations and subcellular localizations according to different experimental and bioinformatic approaches is provided in Supporting Information, Table S1.

Whereas some *E. coli* biological processes such as chemotaxis (Alexander & Zhulin, 2007) and amino acid biosynthesis (Hernandez-Montes *et al.*, 2008) appear to be almost completely understood, knowledge regarding the others such as the biogenesis of the bacterial cell-envelope is constantly increasing in terms of the number of novel components and functionally significant interactions (Ruiz *et al.*, 2008; Scheurwater & Clarke, 2008). Indeed, despite the broad biological implication and clinical significance, the fraction of cell-envelope associated proteins with unknown or poorly described functions approaches 40% in *E. coli* (Fig. 1). Most biochemical studies performed on the cell-envelope to date have been focused on cataloguing individual components rather than understanding the structure as a set of interconnected physical modules (Weiner & Li, 2008). For example, *E. coli* membrane-associated proteins are vastly underrepresented in existing data sets of PPIs. Only 20% of the 1558 binary PPIs derived from low-throughput studies using traditional techniques such as co-immunoprecipitation (co-IP) (Protein co-IP) contained in databases such as DIP (Salwinski *et al.*, 2004), BIND (Bader *et al.*, 2003) or Intact (Kerrien *et al.*, 2007) have at least one interactor tentatively associated with the cell-envelope, and no systematic genome-scale experimental studies of bacterial membrane PPIs have yet been reported, presumably in part because of a lack of suitable high-throughput methods for isolating intact membrane-associated multiprotein complexes. Nevertheless, the existing literature provides valuable information regarding the *E. coli* cell-envelope ‘interactome’.

## The *E. coli* cell-envelope compartments and their associated proteomes

### The IM

The first compartment surrounding the cytoplasm is the IM, which consists of a phospholipidic bilayer that can be spanned by an estimated *c*. 850 TIMPs (Table S1) involved in a broad array of cellular processes, including oxidative phosphorylation, protein secretion and active transport or nutrient uptake. The phospholipidic portion (*c*. 60%) is composed of fatty acids attached to glycerol-3-phosphate that serves as a selective permeable barrier for ions and molecules to pass, either into or out of the cytoplasm. The IM is also the site for the formation of many precursor components that are ultimately exported to form the OM and cell wall.

**Fig. 2:**
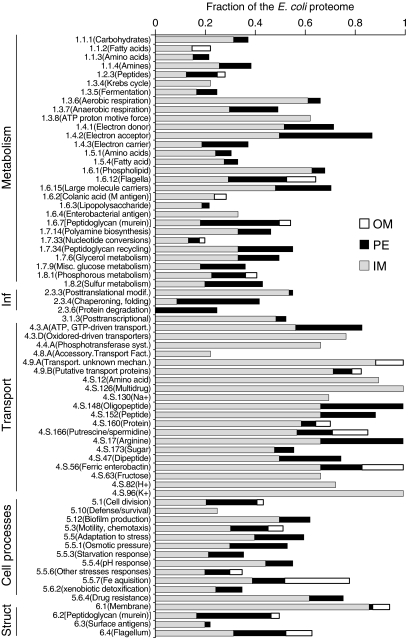
A middle-level functional classification of the *E. coli* cell-envelope-related proteome. The 1179 proteins in the ‘Majority Consensus’ tentatively forming the cell-envelope proteome of *E. coli* K-12 were mapped against the middle-level terms in the hierarchy of functional annotations in the database MultiFun (Serres *et al.*, 2004). Fractions represent the number of cell-envelope proteins for each MultiFun functional category, divided by the total number of *E. coli* proteins in the respective category. Only categories with fractions of tentative cell-envelope proteins >0.2 are shown. Subcellular localization acronyms are described as in Fig. 1. Struct, Structural components; Inf, inner membrane protein folding.

*Escherichia coli* TIMPs possess between 1 and 18 α-helices spanning the IM, each formed by at least 15 amino acid residues (Daley *et al.*, 2005; Punta *et al.*, 2007). TIMPs represent by far the most complete and diverse cell-envelope-related proteome. As shown in Fig. 2, transporters are one of the most populated types of TIMPs (Daley *et al.*, 2005), and knowledge databases such as MultiFun (Serres *et al.*, 2004) and TCDB (Saier *et al.*, 2006) provide detailed descriptions on the types of molecules and mechanisms associated with this class of proteins. Additionally, some TIMPs carry out important metabolic processes such as aerobic and anaerobic respiration and the biosynthesis and transport of most cell-envelope constituents. Likewise, the IM serves as an attachment point for the intracellular protein cytoskeleton (Shih & Rothfield, 2006), and the basal constituents of the flagellum (Berg, 2003).

Because all the OM components are synthesized in the inner leaflet of the IM, they need to be transported across the IM and through the periplasm by diverse molecular machines, including the ATP binding cassette (ABC) transporter MsbA (Doerrler, 2006; Bos *et al.*, 2007). Two TIMPs, YjgP and YjgQ (recently renamed as LptF and LptG), have been suggested to be transmembrane components of this transporter, working together with LptB to extract lipopolysaccharide – a major component of the OM outer leaflet – from the IM *en route* to the OM (Ruiz *et al.*, 2008). Similarly, the TIMPs YfbW and YfbJ (recently renamed as ArnE and ArnF) appear to serve as flippases for lipid A precursors such as undecaprenyl phosphate-α-l-Ara4 (Yan *et al.*, 2007), while the Wzx pathway-related TIMPs are responsible for the translocation of O-antigens (Rick *et al.*, 2003), which are also components of the OM.

Transport of proteins across the cell-envelope compartments is essential for bacterial life and in Gram-negative species it can be mediated by at least six different secretion systems (SSs) (Saier, 2006). In general, proteins to be secreted possess an N-terminal signal peptide sequence allowing recognition by specific SSs. T1SS (Holland *et al.*, 2005), T3SS (Brutinel & Yahr, 2008) and T4SS (Backert & Meyer, 2006) directly translocate proteins from the cytoplasm to the extracellular space. In contrast, T2SS uses two steps: first, a translocation through the IM that can proceed via the SecYEGDF–YidC complex specific for unfolded proteins (Driessen & Nouwen, 2008) or via the twin-arginine system (TatABCE) for folded proteins (Lee *et al.*, 2006). Then, a second step translocates proteins across the OM (see The OM). Most of the *sec*-encoding genes are essential for *E. coli* survival (Baba *et al.*, 2006); in contrast, *tat*-single-gene knock-outs are viable under standard laboratory conditions (Baba *et al.*, 2006). Similarly, T5SS proceeds in two steps, with IM transport performed by an ATP-independent autotransporter coupled coordinately with Sec-mediated passage through the OM (Thanassi *et al.*, 2005).

Several protein SSs, including the recently discovered T6SS (Filloux *et al.*, 2008), as well as T3SS, T4SS and T5SS are involved in bacterial pathogenesis. For example, the classic T3SS is implicated both in the biogenesis of flagella and in the injectosome, a giant molecular syringe that translocates diverse effector proteins into the cytoplasm of host cells promoting pathogenesis or symbiosis (Galan & Wolf-Watz, 2006). Most of these systems have been found in pathogenic *E. coli* strains (Pruitt *et al.*, 2005; Yang *et al.*, 2008).

### The periplasm and cell wall

The periplasm, located between the IM and the OM, contains an estimated *c*. 350 proteins (Table S1), many of which are water-soluble enzymes involved in the biogenesis of the peptidoglycan core layer, the major rigid component of the bacterial cell wall that consists of extensively cross-linked glycan and peptide strands that provide mechanical support. Because the precursors of peptidoglycan are actually synthesized in the cytoplasm, they need to be transported across the IM before assembly of the cell wall. In the betaproteobacterium *Neisseria gonorrhoeae*, it was recently suggested that AmpG or AmpD can participate in peptidoglycan recycling (Garcia & Dillard, 2008), but the major transporter (s) of *de novo* peptidoglycan precursors from the cytoplasm in *E. coli* remains unknown.

As described in the previous section, translocation of proteins through the IM can be mediated by diverse SSs. One of the most abundant components of the periplasm are chaperones (Fig. 2). In the periplasm, protein folding is monitored by DegP, which can serve both as a protease and as a chaperone (Krojer *et al.*, 2008), together with several other core periplasmic factors such as SurA and Skp (Bos *et al.*, 2007). Genetic studies suggest that Skp and DegP act together in a periplasmic chaperone pathway that is functionally redundant with SurA (Rizzitello *et al.*, 2001; Typas *et al.*, 2008). Recently, YaeT was suggested to be functionally related to SurA (Sklar *et al.*, 2007). Other chaperones belonging to the PapD-like superfamily direct the biogenesis of pilus and nonpilus organelles (Behrens, 2003).

Another class of proteins highly populated in the periplasm are lipoproteins, which are covalently attached to either the IM or the OM via modified N-terminal N-acyl-diacylglycerylcysteine phospholipid-containing residues (Tokuda & Matsuyama, 2004; Weiner & Li, 2008). The major portion of bacterial lipoprotein structure typically resides in the water-soluble periplasmic compartment, and all of the known lipoproteins of *E. coli* face the periplasm (Bos *et al.*, 2007). Nonetheless, some authors classify lipoproteins as either components of the IM or the OM proteomes (Molloy *et al.*, 2000; Lopez-Campistrous *et al.*, 2005). Two main functions of lipoproteins are enzymes, such as lytic transglycosylases, which degrade peptidoglycan during the cleavage of the septum, a process necessary for cell division (Scheurwater & Clarke, 2008), or act as structural components, such as members of the Tol-Pal cell-envelope complex, which link the OM to the cell wall and the IM via extensive protein–peptidoglycan and protein–protein interactions (Gerding *et al.*, 2007). Similarly, Lpp, one of the most abundant proteins in *E. coli*, couples the OM to the cell wall (Hirashima *et al.*, 1974; Weiner & Li, 2008). Other lipoproteins, such as Wza, serve as polysaccharide transporters. The specialized database DOLOP (Madan Babu & Sankaran, 2002) classifies known and predicted bacterial lipoproteins according to their putative functions. In general, annotated lipoproteins associated with the OM form a complex with a periplasmic chaperone called LolA, which releases lipoproteins from the IM across the OM assisted by LolB (Yokota *et al.*, 1999; Tokuda & Matsuyama, 2004).

### The OM

The OM is the outermost structure in Gram-negative bacteria, and hence is the interface between the cell and the environment. The canonical model of biological membranes formed by a phospholipid bilayer does not apply to the OM. Instead, the OM is asymmetric, with phospholipids predominantly in the inner leaflet and lipopolysaccharide on the outer leaflet (Nikaido, 2003). Lipopolysaccharide is formed by lipid A and a branched sugar chain anchored to O-antigens that are highly immunogenic and frequently toxic in mammals (Sundararaj *et al.*, 2004; Holst, 2007). Although not essential for *in vitro* culture conditions, lipopolysaccharide is required for infectivity and viability in a living host in *E. coli, Salmonella* sp. and seemingly most other pathogenic bacteria (Ruiz *et al.*, 2008). In contrast to other membrane systems, the OM is quite impermeable to hydrophobic molecules and chemicals, including many antibiotics, in part due to the presence of lipopolysaccharide (Ruiz *et al.*, 2008).

The proteomic diversity of the OM is quite low as compared with the IM counterpart (Fig. 2). In *E. coli*, the OM proteome comprises *c*. 100 TOMPs, with β-barrels spanning the OM, most of which serve as transporters of proteins and small molecules (Molloy *et al.*, 2000; Weiner & Li, 2008). TOMP β-barrels characteristically consists of between 8 and 22 β-strands, each generally longer than 10 residues arranged in an antiparallel configuration (Punta *et al.*, 2007) with hydrophobic residues pointing outward of the barrel (Wimley, 2003) that form mono-, di- and trimeric complexes (Weiner & Li, 2008).

Transport of small solutes (<800 Da) across the OM is mediated mainly by passive diffusion through the water-filled channels of trimeric β-barrel porin proteins. Some of these porins (OmpT and OmpP) are essential to *E. coli* pathogenesis (Hritonenko & Stathopoulos, 2007). Other TOMPs are specific compound transporters, acting coordinately to increase the diversity of transported molecules. For instance, FhuA is involved both in the transport of ferrichrome and, when coupled with TonB, can import siderophore–iron-scavenging complexes back across the OM (Sansom, 1999). In turn, TonB combines with ExbB and ExbD to use the siderophore–iron complex to control the electrochemical gradient across the IM (Sansom, 1999). Additionally, FhuA mediates the transport of diverse antibiotics, exotoxic peptides, and acts as a receptor for certain phages (Braun *et al.*, 2002).

After passing the IM and transiting the periplasm, proteins secreted via the Sec-system (see The IM) can be incorporated into the OM by the Outer Membrane Protein Assembly Complex (Schleiff & Soll, 2005), representing the second part of the T2SS pathway. This complex is formed by at least five Bam-proteins (Wu *et al.*, 2005), including BamA (also called YaeT or Omp85). Depletion of BamA revealed two structurally distinct TOMP subgroups that follow dissimilar folding pathways (Werner & Misra, 2005). In one case, assembly of TOMPs such as TolC, a channel involved in protein and drug secretion, appears to be dependent on BamA (Werner & Misra, 2005), while others such as PulD, one of the so-called secretins (Bayan *et al.*, 2006), appear to be dependent on specific lipoproteins such as PulS for insertion into the OM (Collin *et al.*, 2007). Additionally, the Fim and Pap pathways have been demonstrated to be involved in the translocation of pili components through the OM via the chaperone–usher pathway (Remaut *et al.*, 2008). Also, folding and insertion of TOMPs into liposomes has been reported, suggesting that these processes can take place spontaneously (Kleinschmidt, 2003).

In summary, there is an extensive functional cross-talk between proteins associated with the biogenesis of the cell-envelope and cellular processes occurring across the different subcompartments. In the following sections, we provide a summary of bioinformatic and proteomic tools for deciphering the fundamental mechanistic aspects of the bacterial cell-envelope and defining its associated proteome.

## Deciphering the *E. coli* cell-envelope proteome

### Bioinformatic approaches for investigating the cell-envelope proteome

Some of the features that allow secreted proteins to be directed to specific subcellular compartments, such as the signal peptides, have been well characterized and can be detected on the basis of primary amino acid sequence patterns (Emanuelsson *et al.*, 2007). Additionally, given the hydrophobic nature of biological membranes, TIMPs and TOMPs characteristically show highly hydrophobic regions. This attribute allowed Kyte & Doolittle (1982) to develop the classical hydropathicity index, used by TOPPRED (Claros & von Heijne, 1994), one of the earlier predictors of TIMP α-helices. Furthermore, many proteins found at specific subcellular compartments have been found to possess distinctive amino acid compositions useful for predicting protein localization (Cedano *et al.*, 1997). In addition, Nair & Rost (2002b) noted that both the sequence identity and the secondary structure of proteins can serve as useful predictors of compartmentalization. In parallel, these authors attempted to circumvent some of the inconsistencies by elucidating suitable sequence motifs using LOCkey (Nair & Rost, 2002a), an algorithm to infer subcellular localization using keyword annotations from the protein knowledge base SWISS-PROT (Boeckmann *et al.*, 2003). Modern programs, as elaborated below, incorporate modified versions of these earlier algorithms (Gardy & Brinkman, 2006; Punta *et al.*, 2007) not only to predict individual protein features such as α-helices, β-barrels and signal peptides but also to infer the global pattern of subcellular localization of bacterial proteins on a genomic scale (Table 1).

**Table 1:** Data sources of known and predicted protein subcellular localization analyzed in this study

Type of data source or program	Subcellular localization	References†	Version‡	Batch§
Predictors of α-helix topology				
MEMSAT3	IM	Jones (2007)	3.0	S
Phobius (PolyPhobius)	IM	Kall *et al.* (2004)	–	M
ConPredII	IM	Arai *et al.* (2004)	2005	M
TMHMM	IM	Krogh *et al.* (2001)	2.0	M
HMMTOP	IM	Tusnady & Simon (1998)	2.0	M
Discriminators of OM proteins and predictors of β-barrel topology				
BOMP	OM^*^	Berven *et al.* (2004)	–	M
TMB-Hunt	OM^*^	Garrow *et al.* (2005)	–	S, M
TMBETADISC-RBF	OM^*^	Gromiha *et al.* (2007a, b)	–	M, G
TMBETA-NET	OM^*^ and OM^**^	Gromiha & Suwa (2005)	–	S
PRED-TMBB	OM^**^	Bagos *et al.* (2004)	–	S
PROFtmb	OM^*^ and OM^**^	Bigelow *et al.* (2004)	–	M (10)
Predictors of signal peptides¶				
DOLOP∥	LP	Babu *et al.* (2006)	1.0	G
TatP	PE, EC	Bendtsen *et al.* (2005)	1.0	M
SignalP	PE, OM, EC	Bendtsen *et al.* (2004)	3.0	M
LipoP	LP	Juncker *et al.* (2003)	1.0	M
Predictors of global subcellular localization				
Majority consensus††	CY, IM, PE, OM, EC	This study	NA	NA
Gneg-PLoc	CY, IM, PE, OM, EC, FB, FG, NC	Chou & Shen (2006)	2.5	S‡‡
CELLO II	CY, IM, PE, OM, EC	Yu *et al.* (2006)	2.5	M
PSORTb	CY, IM, PE, OM, EC	Gardy *et al.* (2005)	2.0	M, G
P-CLASSIFIER	CY, IM, PE, OM, EC	Wang *et al.* (2005)	2005	M (100)
Proteome Analyst	CY, IM, PE, OM, EC	Lu *et al.* (2004)	2.5	M, G
Proteomic studies				
Zhang *et al.*	CY, IM, PE, OM	Zhang *et al.* (2007)	NA	NA
Lopez-Campistrous *et al.*	CY, IM, PE, OM	Lopez-Campistrous *et al.* (2005)	NA	NA
Daley *et al.*	IM	Daley *et al.* (2005)	NA	NA
Mori and colleagues	M	Unpublished§§	NA	NA
Molloy *et al.*	OM	Molloy *et al.* (2000)	NA	NA
Knowledge databases				
TOPDB	IM, OM	Tusnády *et al.* (2008)	2007	M
EcoCyc	CY, IM, PE, OM, EC, LP	Karp *et al.* (2007)	11.6	M
Riley *et al.*	CY, IM, PE, OM, LP	Riley *et al.* (2006)	NA	M
ePSORTdb	CY, IM, PE, OM, EC	Rey *et al.* (2005a)	2.0	M
MultiFun	CY, IM, PE, OM, EC	Serres *et al.* (2004)	2007	M
CCDB	CY, IM, M, PE, OM, EC, LP	Sundararaj *et al.* (2004)	2006	M
Uniprot	CY, IM, M, PE, OM, EC, LP	Boeckmann *et al.* (2003)	55.5	M, G
DOLOP	LP	Madan Babu & Sankaran (2002)	2005	M

† Corresponding websites are provided in Table S1.

‡ Some programs or databases do not provide a version other than referring to the year of the last webpage update. In all these cases, the data were collected in May, 2008. NA, not available; –, no version availability.

§ Corresponding websites allow the submission of multiple sequences (M), provides precomputed genomic results (G), or allow only submission of single sequences (S). PROFtmb and P-CLASSIFIER allows submitting of up to 10 and 100 sequences per run, respectively. TMB-Hunt allows submitting of multiple sequences if a homology-based step is turned-off.

¶ Some α-helix programs such as Phobius, and Conpred II has its own signal peptide predictors.

∥ DOLOP detects potential lipoprotein features at the NH_3_-terminus of protein sequences (not necessarily signal peptides). Also provides a list of experimentally verified lipoproteins.

†† The ‘Majority Consensus’ is not a predictor itself, is just the integration of results from the four global predictors of subcellular localization with predictions available in batch mode (PSORTb, Proteome Analyst, CELLO II and P-CLASSIFIER).

‡‡ Gneg-PLoc provides precomputed results for proteins that have no subcellular localization annotations or annotated with uncertain terms such as ‘probable’, ‘potential’, ‘likely’, or ‘by similarity’ in Swiss-Prot.

§§ http://ecoli.naist.jp/GFP/gfp_top.jsp

CY, cytoplasmic; PE, periplasmic; OM^*^, discriminator of outer membrane β-barrels; OM^**^, β-barrel topology predictor; M, membrane (undefined if IM or OM); EC, extracellular; FB, fimbriae; FG, flagellum; NC, nucleoid; LP, lipoproteins (might be part of different cell-envelope compartments).

#### Statistical parameters to evaluate the performance of predictors of subcellular localization

The performance of bioinformatic prediction tools can be evaluated by rigorous statistical measures. One common strategy is cross-validation of predictions against an annotated reference data set or the so-called ‘gold standard’. We extended a seminal performance evaluation (Gardy & Brinkman, 2006) of different predictors of either protein global subcellular localization or specific protein features (e.g. α-helices and β-barrels), to include novel methods (and updated versions), and to control some specific parameters of feature-based predictors. To this end, we compared the predictions from several methods against a gold standard comprising 299 sequences of proteins with well-documented subcellular localization (Gardy & Brinkman, 2006), from different Gram-negative species, including *E. coli*. The gold standard is ensured to contain no close relatives within the training sets of the methods being evaluated (cutoff=80% identity) and includes 145 proteins from the cytoplasm, 69 from the IM, 38 from the OM, 29 from the periplasm and 18 extracellular. The full set of 299 sequences was inputted to each predictor and the results (Table S2) were contrasted against the actual localization of the proteins.

Three standard performance measures were calculated: (i) ‘sensitivity’ or the ability of the predictor to obtain correct predictions (true positives), (ii) ‘precision’ or the capacity of the predictor to distinguish between true positives and incorrect hits and (iii) the Matthews Correlation Coefficient, which provides an overall measure of the predictor performance (see Table 2 footnote for details). We then extended our analysis based on the fact that different methods can display a high precision but low sensitivity for a common task. For example, in the case of prediction of cytoplasmic proteins, sensitivity can still be limited and hence integration of different highly precise methods can result in a larger set of accurate results than any individual approach. Hence, we decided to examine the ‘Agreement’ between the various predictors using the full *E. coli* proteome as a reference (Table S1). Our goal was not only to determine the common hits for each compartment shared by the various methods but also to determine any tendencies among the disagreements between the programs. To this end, we used a simple formula to determine the fraction of common predictions between pairs of methods: 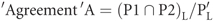
where (P1∩P2)_L_ represents the number of common hits between two predictors (P1 and P2) for a given subcellular location (L). While P′_L_ corresponds to the total number of predictions from the method with a lower coverage for that particular compartment. This coverage normalization was necessary to avoid underestimating effectiveness because comparison of the results from the bioinformatic tools against knowledgebases and low-throughput experimental studies achieved incomplete coverage of the proteome. *A* value of *A*=1 means that all (100%) of the proteins predicted by the method P1 as belonging to a subcellular location L were predicted to be in the same location by the method P2. In change, a value of *A*=0 means that there are no common hits between the two methods for a particular subcellular location. Moreover, to evaluate the ‘Agreement’ between different predictors of global subcellular localization and those detecting specific features, we split the predictions from the former according to cellular compartment, and grouped the predictions of IM proteins with predictors of TIMP α-helices (Table 1) and the predictions of OM proteins with predictors of TOMP β-barrels. The results from both sets of analyses, ‘Performance’ (sensitivity and precision) and ‘Agreement’ (common hits), are reported in the following sections devoted to each type of predictor.

**Table 2:** ‘Performance’ comparison of predictors of global protein subcellular localization, α-helices (TMHs) and β-barrels (TMBs)†

Predictor‡	TP	FP	FN	TN	Precision (%)	Sensitivity (%)	MCC
Cytoplasmic							
Majority Consensus^*^	131	3	14	151	97.76	90.34	0.89
Proteome Analyst^*^	119	7	26	147	94.44	82.07	0.78
CELLO II^*^	135	24	10	130	84.91	93.10	0.78
PSORTb^*^	108	2	37	152	98.18	74.48	0.76
P-CLASSIFIER^*^	135	29	10	125	0.82	0.93	0.75
GnegPLoc^*^	132	50	6	95	72.53	95.65	0.64
Inner membrane and α-helices							
Proteome Analyst^*^	65	10	4	220	86.67	94.20	0.87
Majority Consensus^*^	54	1	15	229	98.18	78.26	0.85
Phobius [≥1 TMHs]§	55	2	14	228	96.49	79.71	0.85
PSORTb^*^	53	2	16	228	96.36	76.81	0.83
TMHMM [≥2 TMHs]	43	1	26	229	97.73	62.32	0.74
GnegPLoc^*^	48	6	19	210	88.89	71.64	0.74
TMHMM [≥1 TMHs]	53	12	16	218	81.54	76.81	0.73
Phobius [≥2 TMHs]	43	2	26	228	95.56	62.32	0.72
ConPredII [≥2 TMHs]	43	2	26	228	95.56	62.32	0.72
CELLO II^*^	43	2	26	228	95.56	62.32	0.72
P-CLASSIFIER^*^	41	2	28	228	0.95	0.59	0.70
MEMSAT3 [≥2 TMHs]	42	3	27	227	93.33	60.87	0.70
ConPredII [≥1 TMHs]	56	21	13	209	72.73	81.16	0.69
HMMTOP [≥2 TMHs]	42	9	27	221	82.35	60.87	0.64
HMMTOP [≥1 TMHs]	54	76	15	154	41.54	78.26	0.38
MEMSAT3 [≥1 TMHs]	69	230	0	0	23.08	100.00	NA
Periplasmic							
Majority Consensus^*^	21	6	8	264	77.78	72.41	0.72
PSORTb^*^	17	2	12	268	89.47	58.62	0.70
Proteome Analyst^*^	21	13	8	257	61.76	72.41	0.63
CELLO II^*^	22	22	7	248	50.00	75.86	0.57
P-CLASSIFIER^*^	19	21	10	249	0.48	0.66	0.50
GnegPLoc^*^	8	7	20	248	53.33	28.57	0.34
Outer membrane and β-barrels							
PSORTb^*^	30	0	8	261	100.00	78.95	0.88
Proteome Analyst^*^	30	0	8	261	100.00	78.95	0.88
Majority Consensus^*^	29	0	9	261	100.00	76.32	0.86
PRED-TMBB [≥3 TMBs]¶	26	6	12	117	81.25	68.42	0.68
PROFtmb [≥3 TMBs]	19	0	19	123	100.00	50.00	0.66
PROFtmb [≥2 TMBs]	19	0	19	123	100.00	50.00	0.66
BOMP [blast allowed]	20	1	18	122	95.24	52.63	0.65
GnegPLoc^*^	18	4	15	246	81.82	54.55	0.63
TMBETA-NET [≥3 TMBs]	31	24	7	99	56.36	81.58	0.56
TMBETA-NET [≥2 TMBs]	31	24	7	99	56.36	81.58	0.56
CELLO II^*^	21	10	17	251	67.74	55.26	0.56
PRED-TMBB [≥2 TMBs]	35	40	3	83	46.67	92.11	0.51
P-CLASSIFIER^*^	20	13	18	248	0.61	0.53	0.51
TMB-Hunt	18	10	20	251	64.29	47.37	0.50
TMBETADISC-RBF	28	36	10	225	43.75	73.68	0.49
Extracellular							
Majority Consensus^*^	7	0	11	281	100.00	38.89	0.61
Proteome Analyst^*^	10	8	8	273	55.56	55.56	0.53
PSORTb^*^	5	0	13	281	100.00	27.78	0.52
CELLO II^*^	8	12	10	269	40.00	44.44	0.38
P-CLASSIFIER^*^	6	13	12	268	0.32	0.33	0.28
GnegPLoc^*^	4	6	13	260	40.00	23.53	0.27

† A set of 299 proteins from Gram-negative bacterial species was used as reference gold standard, with exception of PRED-TMBB, PROFtmb and TMBBETA-NET, which allow the submission of only one or few sequences at a time. For these programs, we randomly selected a subset of 161 proteins, restricting the subsets of CY, IM and OM to 38 proteins each. Sixteen out of the 299 proteins predicted by Gneg-PLoc as part of nucleoid, flagellum or fimbriae were excluded from Gneg-PLoc performance analysis. All predictions were run in September 2008 (see Table S2 for details).

TP, true positives; FP, false positives; FN, false negatives; TN, true negatives. Precision = TP / (TP+FP); Sensitivity = TP / (TP+FN). Sections for each subcellular localization in this table show predictors from higher to lower Matthews Correlation Coefficient (MCC), using the following formula: 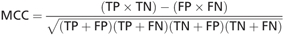

‡ Predictors of global subcellular localization are denoted by (^*^).

§ Predictors of TMHs and TMBs were analyzed twice to filter the minimal number of trans-membrane elements required to count as a true hit (shown in square parentheses).

¶ PRED-TMBB includes three methods; only the Viterbi method is shown here. The other two methods resulted in similar performance (Table S2).

#### Predictors of protein global subcellular localization

##### Predictors using support vector machine (SVM) algorithms

SVMs are supervised learning methods used to classify data into different subgroups. In this case, using a ‘training’ data set, the SVM algorithm attempts to determine whether a protein belongs or not to a single specific subcellular localization. Two of the most accurate predictors of this type (Gardy & Brinkman, 2006) are CELLO (Yu *et al.*, 2004) and P-CLASSIFIER (Wang *et al.*, 2005), which provide a tentative subcellular localization for each inputted sequence (see Table 1). CELLO uses a combination of five SVMs to look for different sequence features such as amino acid composition and sequence specific motifs. In an updated version, CELLO II incorporates a homology-based step that increases the performance of the program (Yu *et al.*, 2006). P-CLASSIFIER uses 15 SVMs in which protein sequence fragments are examined considering different physicochemical-based groupings of similar amino acids, and was developed expressly for Gram-negative species.

The data used to train the SVMs, and predictors in general, greatly influence the performance of the method. Both CELLO and P-CLASSIFIER are based on ePSORTdb (Rey *et al.*, 2005a), a database of proteins with well-documented subcellular localization. CELLO II discarded the proteins annotated in ePSORTdb to have multiple subcellular localizations, and it also incorporates additional sequences from the SWISS-PROT database (Boeckmann *et al.*, 2003) to increase sensitivity for eukaryotic proteins. Comparing the performance reported for CELLO (Gardy & Brinkman, 2006) and CELLO II against the gold standard set of 299 proteins (Table 2), we found that a single category (extracellular) showed increased performance (*c*. 10%), while the others remained comparable.

##### Predictors using multicomponent analytical pipelines

This class of predictors uses a series of analytical tools (called modules) to assign likelihoods for a given protein to be located at a specific compartment. psortb (Gardy *et al.*, 2005) incorporates a number of modules, each devoted to a specific prediction task, including: homology-based predictions (blast against epsortb), a transmembrane α-helix predictor called hmmtop (Tusnady & Simon, 1998) (described in Predictors of TIMP α-helices), a signal peptide predictor, a series of frequent subsequence-based SVMs and a motif- and profile-matching module. The aggregate produced by psortb has been reported as one of the most precise ensemble methods not only among multicomponent pipelines but also in subcellular localization predictions in general (Gardy & Brinkman, 2006; Zhou *et al.*, 2008). Consistent with this, we found that psortb accurately predicted proteins belonging to the OM, potentially TOMPs (Table 2), even outperforming specialized β-barrel predictors (described in Discriminators of TOMP's and predictors of β-barrel's topology). cpsortb (Rey *et al.*, 2005a, b) is a database companion to psortb that contains precomputed subcellular localization predictions for entire proteomes including that of *E. coli*.

psortb developers have highlighted their goal of emphasizing precision at the expense of sensitivity. In that sense, psortb returns an ‘unknown’ designation for certain proteins if a confident prediction was not generated or a potential ‘dual localization’ assignment if substantive conflicting evidence is inferred. In general, *c*. 60–70% of proteins in prokaryotic genomes can be assigned to a specific subcellular compartment by psortb (Gardy & Brinkman, 2006). In the case of *E. coli* W3110, psortb predicts a subcellular localization for *c*. 64% of the entire proteome (ORFeome), while just over 1500 proteins currently lack predictions (i.e. unknown) and a further 57 are predicted with ‘potential dual’ localizations. In contrast, an SVM-based algorithm such as CELLO II and P-CLASSIFIER return a prediction for every inputted sequence and hence their sensitivity can be higher, but precision generally appears to be lower (Table 2).

##### Predictors using lexical (keyword) annotations

As mentioned before, a completely different type of predictor uses keywords of protein preexisting annotations from databases such as SWISS-PROT to assess subcellular localization. In 2002, LOCkey (Nair & Rost, 2002a), one of the first predictors of this class, was reported with a remarkable precision of 82%, albeit with a sensitivity of <50%, presumably due in part to a lack of existing functional annotations. Later, Chou & Cai (2003) demonstrated that by combining the complementary information present in gene ontology (GO) annotations (Ashburner *et al.*, 2000), functional domain databases and sequence-specific features, the success rate of subcellular localization prediction can be increased up to 94.7%.

Proteome Analyst (Lu *et al.*, 2004) uses machine-learned classifiers to analyze keywords derived from various annotation databases, including GO and GeneQuiz (Andrade *et al.*, 1999), predicting diverse properties for each inputted protein sequence, including subcellular localization and molecular function. Proteome Analyst uses a Naïve Bayes classifier and a graphical interactive interface to increase transparency in terms of the basis for particular predictions, improving user confidence as to why a particular subcellular localization is chosen over others when conflicting outputs coexists. PA-GOSUB (Lu *et al.*, 2005) is a companion database to Proteome Analyst containing predictions of the molecular functions and subcellular localization for a selection of genomes from the three cellular domains that can be extended upon request. Additionally, Proteome Analyst can create a custom classifier to predict a new property based on labeled training data. Like PSORTb, Proteome Analyst does not retrieve results for every sequence, but can predict a subcellular localization for *c*. 88% of the *E. coli* ORFeome and appears to be particularly accurate in determining IM proteins, potentially TIMPs (Table 2).

Our ‘Agreement’ analysis shows that 88 proteins predicted as cytoplasmic by psortb and at least one of the SVM-based global predictors are, in contrast, predicted as extracellular (32) or periplasmic (56) by Proteome Analyst (Table S1). Additionally, using TatP, we failed to detect periplasmic or extracellular signal peptide sequences and currently GO (v36.0) indicates a cytoplasmic localization for only 16 of them, with most of the others having no assignment. This suggests that Proteome Analyst inherited these annotations from older versions of GO or GeneQuiz, implying further that the incorporation of basic sequence-based filters could markedly improve the performance of Proteome Analyst and other keyword-based predictors.

**Fig. 3:**
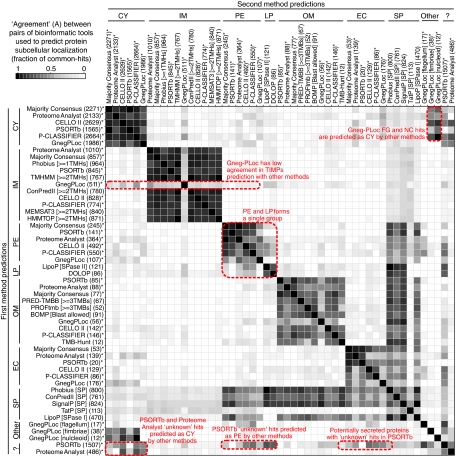
‘Agreement’ analysis between pairs of bioinformatic predictors of protein subcellular localization. The 4220 proteins forming the *E. coli* K-12 proteome were subjected to prediction of global subcellular localization (^*^) and specific features (α-helices, β-barrels and signal peptides) by different computational methods. Each square in the matrix represents the number of proteins predicted to be located in a given compartment by any two predictors (P1 and P2). Results from P1 are plotted on the *x*-axis, while predictions of P2 are plotted on the *y*-axis. The number of predicted proteins for each subcellular location by each method is shown in parentheses. The darker the square intersecting any two methods, the higher the ‘Agreement’ between them (see section ‘Statistical parameters to evaluate the performance of predictors of subcellular localization’ for details). Major discrepancies between methods are highlighted in red frames. TIMP α-helix predictors were evaluated for one or more helices (≥1 TMHs) and for two or more helices (≥2 TMHs); only the option with a higher ‘Performance’ (Table 2) is shown. CY, cytoplasmic; SP, signal peptide;‘?’ refers to proteins with no predicted localization. Other subcellular localization acronyms are described as in Fig. 1. Subcellular localization predictions and ‘Agreement’ values used to construct this plot are available in Table S1.

Gneg-PLoc (Chou & Shen, 2006) is one of the first algorithms that uses a type of classifier called Neural Network (NN) to integrate lexical annotations (such as GO terms) with protein amino acid composition to predict protein subcellular localization. In addition to the five commonly predicted compartments (Table 1), Gneg-PLoc is able to predict flagellum, fimbrium and nucleoid proteins. Despite these interesting features, our performance evaluation positions for the Gneg-Ploc was below other global subcellular localization predictors (Table 2), even when restricting the cross-validation to the former five compartments. The ‘Agreement’ comparison showed that Gneg-PLoc has only 40% of common hits with other methods in predicting IM proteins (Fig. 3). About 80% of the nucleoid predictions from Gneg-PLoc correspond to proteins predicted as cytoplasmic by other methods, while *c*. 40% of putative fimbrial proteins correspond to cytoplasmic hits by other methods. In fact, some of the latter, such as DnaT, involved in DNA replication, and SufB and SufD, involved in the iron–sulfur cluster assembly, have clear cytoplasmic roles. While our ‘Agreement’ analysis of Gneg-PLoc was restricted to the 2903 precomputed *E. coli* protein predictions available in the Gneg-PLoc web server, which unfortunately only allows prediction of one sequence at a time, hence the generality of these findings to the entire *E. coli* ORFeome was not assessed, these results suggest that some Gneg-PLoc inferences are markedly different from other predictors.

##### A ‘Majority Consensus’ improves the prediction of global subcellular localization

Our ‘Agreement’ analysis shows that 671 out of the 1507 proteins deemed without predicted subcellular localization (unknown) by psortb were nevertheless predicted as cytoplasmic by both Proteome Analyst and at least one of the SVM-based predictors (CELLO II and/or P-CLASSIFIER) (bottom part of Fig. 3), while another 94 proteins were predicted as PE, 48 as IM, 24 as EC and three as OM by at least two different method types (bottom part of Fig. 3). Conversely, 152 out of the 486 proteins without a confident score by Proteome Analyst were predicted as cytoplasmic by psortb and at least by one of the SVM-based methods, whereas another 24 were predicted as IM and one as OM by two different method types. Because psortb, Proteome Analyst and the SVM-based methods have marked different methodological bases for generating predictions, we consider that the common hits represent robust information that could potentially be incorporated into multicomponent pipelines when ‘unknown’ or ‘low-confidence’ hits are obtained.

In light of these findings, we integrated the results from the four predictors of global subcellular localization with the highest performance (psortb, Proteome Analyst, CELLO II and P-CLASSIFIER) by a simple majority rule, wherein each type of predictor has one vote (i.e. the two SVM-based predictors vote together once). This provided inferences for a set of 3503 proteins, which we call the ‘Majority Consensus’, representing *c*. 83% of the *E. coli* proteome that can be assigned to a subcellular localization by at least two types of predictors of global subcellular localization. This included 2271 proteins predicted as cytoplasmic and 1179 predicted to form the *E. coli* cell-envelope proteome (857 IM, 245 PE and 77 OM), and another 53 as likely exported (extracellular) (Table S1).

As noted in Table 2, the ‘Majority Consensus’ results in increased sensitivity and precision as compared with single methods for most of the compartments, strongly suggesting that the creation of a meta-server that allows for the submission of multiple sequences to diverse subcellular location predictors and integration with ‘a single click’ is desirable for both convenience and improved performance.

The remaining 17% (717 proteins) not included in the ‘Majority Consensus’ could represent cases with multiple dynamical subcellular localizations – for example, changing compartmentalization depending on the cell-growth conditions, as predicted for 24 out of the 717 by PSORTb, or they could simply represent proteins that are difficult to assign computationally to a single compartment using the current strategies. Lipoproteins, for example, are not currently grouped in a single subcellular localization by global predictors. Indeed, we found that 35 of the 86 putative lipoproteins of *E. coli* predicted by DOLOP (Babu *et al.*, 2006) are part of those 717 ‘nonconsensus’ proteins. Conversely, 22 of the 86 putative lipoproteins (*c*. 25%) are predicted to be periplasmic in the ‘Majority Consensus’ (central part of Fig. 3), while 11 are assigned to IM and OM apiece. Lipoproteins are covalently linked to either the IM or the OM and thus some authors consider them as integral parts of these respective compartments (Molloy *et al.*, 2000; Lopez-Campistrous *et al.*, 2005). However, the fact that the main component of the lipoprotein structure resides in the periplasm may be the reason why certain methods do not group them into a single compartment.

##### Summary of predictors of global subcellular localization

The ‘Performance’ and ‘Agreement’ values of predictors of global subcellular localization vary depending on the compartment analyzed. For instance, Proteome Analyst leads the TIMP predictions, while psortb scores highly in TOMP predictions. Moreover, the ‘Majority Consensus’ outperformed all the separate methods when predicting cytoplasmic, periplasmic and extracellular proteins (Table 2).

In general, the highest performance was found in predictions of cytoplasmic proteins (Table 2). This is reflected in a high average ‘Agreement’ among predictors for this compartment (*A*=0.88), followed by the IM (*A*=0.75), the PE (*A*=0.73) and the OM (*A*=0.67). In contrast, the predictions corresponding to the EC proteins show an ‘Agreement’*A*=0.63. These results coincide with previous reports showing that EC proteins are the most difficult population to be modeled (Rey *et al.*, 2005b; Gardy & Brinkman, 2006; Zhou *et al.*, 2008), possibly due to lack of suitable-sized training data sets. LocateP (Zhou *et al.*, 2008) is a recently developed multicomponent predictor of global subcellular localization (in a manner similar to psortb) for Gram-positive bacteria. LocateP developers emphasized their efforts by collecting an experimentally derived protein training set particularly enriched in *bona fide* extracellular protein signal peptides. Accordingly, the predictions of LocateP for this location outperform all other methods (Zhou *et al.*, 2008), including psortb and CELLO. On this basis, the incorporation of more powerful signal peptide classifiers along with larger training data sets could markedly increase the performance of global subcellular predictors for the extracellular proteins of Gram-negative bacteria as well.

#### Predictors of specific protein features

##### Predictors of TIMP α-helices

A number of programs recently reviewed in Punta *et al.* (2007) have been developed to determine TIMP α-helices and TOMP β-barrels (Table 1). In both scenarios, hidden Markov model (HMM)-based methods have been found to outperform those involving SVMs or NNs (Bagos *et al.*, 2005; Punta *et al.*, 2007). All these methods look for specific sequence motifs, profiles or biochemical properties associated with a particular group of proteins tentatively with a similar topology. A common caveat in earlier TIMP α-helix predictors such as HMMTOP (Tusnady & Simon, 1998) and tmhmm (Krogh *et al.*, 2001) is that signal peptides tend to be erroneously assigned as a potential α-helix (Lao *et al.*, 2002). For this reason, some studies (Daley *et al.*, 2005) prefer to discard predictions based on a single potential α-helix to avoid false positives. In the same way, some predictors of α-helices incorporate their own signal peptide detectors. For instance, Phobius (Kall *et al.*, 2004) uses an HMM- and homology-based strategy to predict TIMP topologies and signal peptides. Similarly, conpred ii (Arai *et al.*, 2004) combines the outputs from several TIMP topology and transmembrane region predictors to create a unified model reportedly more accurate than the parental individual methods (Arai *et al.*, 2004), and also allows to activate a signal peptide detector. In contrast, memsat3 (Jones, 2007) integrates a signal peptide detector and evolutionary information (homology) to construct TIMP topological models.

Our performance results (Table 2) coincided with previous assessments (Gardy & Brinkman, 2006; Jones, 2007) suggesting Phobius as one of the most accurate predictors of α-helices. Surprisingly, the overall ‘Performance’ of Phobius is better when all predictions (including sequences with one or more potential α-helices) are considered as positive hits. In contrast, all the other α-helix predictors mentioned above showed improved performance when filtering hits with a single predicted α-helix. We note that memsat and conpredii (with its signal peptide detector activated) show a ‘Performance’ (Table 2) and ‘Agreement’ (Table S1) similar to tmhmm and hmmtop, suggesting that while these methods show a good precision to ‘draw’ topological models of TIMPs, they have difficulties in discriminating between TIMPs and non-TIMPs, presumably because a number of *bona fide* signal peptides are escaping their signal peptide discriminators.

Other strategies to discriminate TIMPs based on free-energy models have been derived solely from experimental data showing that protein loops connecting TIMP transmembrane segments are enriched in positive-charged amino acids, also termed the ‘positive-inside’ rule (Bernsel *et al.*, 2008).

##### Discriminators of TOMPs and predictors of β-barrel's topology

Predictors assessing the occurrence of TOMP features can be divided into two categories: (i) the ‘discriminators’ of TOMPs from non-TOMPs, commonly based on amino acid composition and/or sequence motifs, and (ii) the TOMP β-barrel's topology predictors. Among the second category, PRED-TMBB (Bagos *et al.*, 2004) is an HMM-based method with high accuracy (Table 2) (Bagos *et al.*, 2005). Unfortunately, to our knowledge, the current web servers of β-barrel's topology predictors, including PRED-TMBB, limit users to submit only one or a few sequences at a time and no precomputed predictions are available. PROFtmb (Bigelow *et al.*, 2004) is another β-barrel's topology predictor based on HMMs following closely the performance of PRED-TMBB (Table 2), but the server currently allows submission of 10 or less query sequences.

Conversely, BOMP (Berven *et al.*, 2004) is a discriminator of TOMPs and non-TOMPs that first searches for a C-terminal pattern typically for many well-characterized β-barrels and then calculates a TOMP likelihood score based on the overall amino acid composition of the input sequence. BOMP can incorporate a homology step that increases its ‘Performance’ (Berven *et al.*, 2004) and submissions can be performed for multiple sequences. Similarly, TMB-Hunt (Garrow *et al.*, 2005) is a discriminator of TOMPs and non-TOMPs that uses total amino acid composition criteria. Although TMB-Hunt results are significantly enhanced using homology information (Garrow *et al.*, 2005), this option restricts the submission of single sequences at a time. TMBETADISC-RBF (Ou *et al.*, 2008) and the companion precomputed database TMBETA-GENOME (Gromiha *et al.*, 2007a, b) are other TOMP discriminators, while TMBETA-NET (Gromiha *et al.*, 2005) couples a discriminator with a predictor of β-barrel's topology showing better performance than TMBETADISC-RBF (Table 2), but currently searches are constrained to single sequences.

The performance of various β-barrel's topology predictors has been evaluated by Bagos *et al.* (2005) using a data set of 20 previously defined TOMP β-barrels, and it was reported that most methods perform better when only TOMP β-barrel domains are used for prediction, rather than the full-length sequences. The authors also provide a metaserver (ConBBPRED) that reportedly outperforms single methods (Bagos *et al.*, 2005). While our results (Table 2) coincide with previous findings that HMM-based predictors outperform other predictor types (i.e. NNs and SVMs), we consider that β-barrel's topology predictors could be more useful tools if they allowed for submission of multiple sequences and/or provided precomputed runs, such as commonly studied genomes such as *E. coli*. Conversely, the user (or ideally the algorithm itself) could incorporate β-barrel discriminators – which are presumably computationally less expensive than β-barrel topology predictors – to filter out highly probable non-TOMPs when large sets of sequences are submitted.

##### Predictors of signal peptides

A number of signal peptide predictors for proteins exported by the various cell-envelope SSs have recently been reviewed (Zhang & Henzel, 2004; Emanuelsson *et al.*, 2007). A commonly used suite of signal peptide detectors includes LipoP (Juncker *et al.*, 2003), SignalP (Bendtsen *et al.*, 2004) and TatP (Bendtsen *et al.*, 2005). LipoP is an HMM-based predictor of lipoprotein signal peptides in Gram-negative bacteria, while SignalP combines several NNs with HMMs to detect signal peptides in both bacteria and eukaryotes and also provides a prediction of cleavage sites. TatP combines two NNs to predict Tat-based signal peptides in proteins that are exported in a folded format across the IM (see The IM). The performance of these methods is reportedly high (>90%) for Gram-negative bacteria (Emanuelsson *et al.*, 2007), suggesting that their incorporation as modules of predictors of global subcellular localization or discriminators of specific features (for instance in α-helix predictors) would be of clear benefit.

##### Summary of specific feature predictors

In general, predictors of global subcellular localization outperform methods assessing specific protein features (i.e. α-helices, β-barrels and signal peptides), with the exception of Phobius, whose signal peptide predictions result in more accuracy than any other method for predicting exported proteins (Gardy & Brinkman, 2006). This could be one of the reasons why Phobius is also one of the most accurate predictors of TIMPs (Table 2) as it can discriminate between signal peptides and TIMPs with few helices. The strategy followed by ConPredII combining predictions from different methods is reported (Arai *et al.*, 2004) to increase its effectiveness as compared with the separate methods; however, our ‘Performance’ and ‘Agreement’ analyses suggest that the ConPredII's signal peptide detector limits the precision of this program, and similarly for memsat3.

On the other hand, β-barrel topology predictors outperform the TOMP discriminators (Table 2), although our analysis was limited to a subset of sequences from the 299 gold standard (see Table 2 footnote) because the topology tools limit the number of submitted sequences. In that sense, databases of precomputed runs for commonly studied genomes are particularly useful. Conversely, discriminators of TOMPs offer a good choice to prefilter sequences before β-barrel topology assessment on a large scale. For example, BOMP allows the submission of multiple sequences and shows a ‘Performace’ comparable with some β-barrel topology predictors.

#### Sequence alignment tools dealing with transmembrane regions

While the bioinformatic tools described in previous sections have been developed explicitly to identify cell-envelope-related proteins, other methods have been inherited from studies of the water-soluble proteome and thus need to be used with caution because common amino acid substitution matrices such as BLOSUM and PAM were not developed for transmembrane regions and conventional programs such as blast commonly exclude by default these regions from sequence comparisons because they tend to be highly repetitive (low complexity). Instead, more suitable substitution matrices have been developed for TIMPs. Among them, the SLIM (Muller *et al.*, 2001) index has been reported as the one with the highest accuracy (Punta *et al.*, 2007). Additionally, TM-PSI (Hedman *et al.*, 2002) is a modified version of blast (Altschul *et al.*, 1997) for sequence comparison of transmembrane-containing proteins and can significantly improve detection of evolutionarily related TIMPs. STAM (Shafrir & Guy, 2004) is a sequence alignment program for transmembrane proteins that accounts for different physical properties at various segments of the protein, while PRALINE™ (Pirovano *et al.*, 2008) combines transmembrane region predictors with membrane-specific scoring matrices to enhance multiple sequence alignments.

### Proteomic approaches for investigating the cell-envelope proteome

In this section, we provide a survey of various small- and large-scale experimental methods used over the past decade to decipher the cell-envelope-related proteome of *E. coli* and other microorganisms. These include gel-based and gel-free approaches for separating and identifying proteins associated with various subcellular compartments (Han & Lee, 2006; Poetsch & Wolters, 2008). Because most of these tools share methodological principles with proteomic assays developed to decipher PPIs within the cell-envelope. We also include a discussion of labeling and affinity tagging procedures for isolating and characterizing stable multiprotein complexes, and various techniques for deducing binary interactions, such as the bacterial two-hybrid system (Strauch & Georgiou, 2007), surface plasmon resonance (SPR) (Visudtiphole *et al.*, 2006) and biochemical cofractionation (Tikhonova & Zgurskaya, 2004). For specific details on these methods, readers are urged to look over the recently published reviews (Krause, 2006; Hooker *et al.*, 2007; Poetsch & Wolters, 2008; Weiner & Li, 2008). In section ‘Advances in the proteomic approaches for elucidating the *E. coli* cell-envelope PPIs and protein complexes’, we discuss how the integration of different proteomic approaches can clarify the biological functions of various subunit components of isolated putative membrane complexes.

#### Gel-based separations

##### Conventional two-dimensional (2D)-polyacrylamide gel electrophoresis (PAGE)

Most published proteomic analyses of the *E. coli* cell-envelope have typically been performed by biochemically fractionating mechanically lysed cells, followed by identification of the various components by 2D sodium dodecyl sulfate (SDS)-based 2D-PAGE and/or MS. In some cases, solubilization of proteins associated with different cell-envelope compartments requires the use of strong zwitterionic detergents to improve the performance of 2D-PAGE separation of cell-envelope proteins (Weiner & Li, 2008).

Many of the integral membrane proteins of *E. coli* have been solubilized successfully with detergents and organic solvents. In recent years, the efficacy of 2D-PAGE has been a useful tool for examining bacterial proteomes under different growth conditions (Volker & Hecker, 2005; Han & Lee, 2006; Poetsch & Wolters, 2008). For example, Lopez-Campistrous *et al.* (2005) used 2D-PAGE to compare the *E. coli* proteome under two states of growth (i.e. presence and absence of amino acids) and detected 575 proteins, including 23, whose abundance changed significantly between the two growth conditions. According to the SWISS-PROT version used as a reference in this study, the set of 575 proteins included 368 cytoplasmic factors, 76 TIMPs, 62 TOMPs and 26 periplasmic proteins, with the remaining of unknown localization.

Nonetheless, the natural tendency of proteins to form multimeric complexes can potentially lead to cross-contamination between proteomic extracts from different subcellular localizations. Because TIMPs and TOMPs are difficult to dissolve in aqueous solutions or the extraction buffers commonly used in the purification of cytoplasmic proteins, SDS or other ionic detergents such as sodium cholate or sodium deoxycholate or even nonionic detergents such as 3-[(3-cholamidopropyl)dimethylammonio]-1-propanesulfonate (CHAPS) (Fountoulakis & Gasser, 2003) and Triton X-100 (Kashino, 2003; Dobrovetsky *et al.*, 2005) are generally used for solubilization before electrophoresis. For example, 1%*n*-dodecyl-β-d-maltopyranoside (DDM) has also been used to solubilize the multitopic Na^+^/H^+^ antiporter before 2D-PAGE (Kashino, 2003). Other nonionic detergents such as lauryldimethylaminoxide (LDAO) and octyl-b-d-glucopyranoside (OG) seem to be particularly effective for isolating intact bacterial membrane complexes (Hooker *et al.*, 2007).

Alternatively, organic solvents such as 1 : 1 ratio of chloroform : methanol can be used to extract hydrophobic proteins before 2D-PAGE (Molloy *et al.*, 1999). Likewise, sodium carbonate has been used to extract TOMPs after *E. coli* cells were first broken by French press lysis, resulting in the identification of 21 of 26 putative TOMPs in *E. coli* (as annotated in SWISS-PROT) using matrix-assisted laser desorption/ionization-time of flight (MALDI-TOF) (Molloy *et al.*, 2000). Other well-known TOMPs, such as the abundant Omp-porins (Kustos *et al.*, 2007), have been identified along with hypothetical proteins, such as YbiL and YeaF, using 2D-PAGE (Fountoulakis & Gasser, 2003). Separation efficiency can potentially be further enhanced using free-flow electrophoresis, a versatile preparative system for isolating TIMPs based on charge-to-size ratios in an electric field before 2D-PAGE (Braun *et al.*, 2007). More detailed procedures for solubilizing and separating bacterial membrane proteins are available in Kashino (2003); Weiner & Li (2008).

Quantitation of differential membrane protein abundance across different cell-envelope compartments, or even under different cellular states, can be achieved using fluorescent protein-reactive dyes before 2D-PAGE. For example, the popular commercial difference gel electrophoresis (DIGE) system (Yan *et al.*, 2002) uses charge-matched *N*-hydroxy succinimidyl ester derivatives of the fluorescent cyanine dyes Cy2, Cy3 and Cy5 to enable pre-electrophoretic labeling of control (e.g. cytoplasmic) and experimental (i.e. membrane enriched) samples. The labeled samples are mixed and run in the same gel, with ‘spots’ color intensities and hence protein relative abundance subsequently quantified using imaging analysis software. Using the 2D-PAGE-DIGE approach, the expressions of several TOMPs (e.g. OmpA, OmpF, OmpT and TolC) and periplasmic proteins (e.g. OppA) have been experimentally verified in *E. coli* (Yan *et al.*, 2002).

##### Native 2D-PAGE

Blue Native PAGE (BN-PAGE) is a gel-based charge separation procedure that relies on tight binding of integral membrane protein complexes with the anionic dye Coomassie blue, such that a mobility shift is evident even with putatively intact endogenous membrane complexes. This technique has been suggested as the most versatile and successful gel-based approach for separating solubilized membrane protein complexes from whole-cell protein mixtures according to protein size (Krause, 2006). BN-PAGE is widely used to analyze cyanobacterial and chloroplast membrane-associated proteomes. In *E. coli*, this technique has been used to separate several prominent membrane protein complexes from the IM such as cytochrome *bd* ubiquinol oxidase, leading to the discovery of YhcB as a probable new member of this complex (Stenberg *et al.*, 2005). Also using this technique, the TIMP YidC was shown to associate with the preprotein translocase of *E. coli*, which transiently contacts the transmembrane segments of nascent TIMPs during membrane insertion (van der Laan *et al.*, 2001). Similarly, the TOMP YaeT was likewise shown to interact with other TOMPs in *E. coli* (Kim *et al.*, 2007), while BN-PAGE was used to identify 160 putative membrane proteins from *E. coli*, including 124 proteins related to the IM and OM (Lasserre *et al.*, 2006).

Other advantages of this gel-based approach are that semi-quantitative information can be obtained, while both modified or protein isoforms can be resolved, allowing the relative distribution and processing of cell-envelope proteins to be correlated to a cellular phenotype (Weiner & Li, 2008). However, a major caveat of BN-PAGE is that the anionic dye may disrupt certain protein interactions (Krause, 2006); elution of the complexes from the gel can also be inefficient (Kashino, 2003).

Under certain conditions, membrane protein complexes can be separated using electrophoretic gels based on their intrinsic charge states alone, without prior dye treatment. This method is referred to as colorless native (CN) PAGE. Although the resolution power of this approach is reduced relative to BN-PAGE, this method can potentially preserve weaker interactions. For example, Alami *et al.* (2007) used CN-PAGE to monitor interactions of components of the SecYEGDF secretory system of *E. coli*. To enhance sensitivity, however, both BN-PAGE and CN-PAGE in parallel are suggested (Krause, 2006).

#### Nongel-based proteomic screening approaches

Two-dimensional-PAGE analysis often fails to detect integral membrane proteins (Santoni *et al.*, 2000). HPLC coupled to tandem MS (LC-MS) is a complementary, versatile and sensitive gel-free proteomic technique for sequencing large numbers of proteins present in a complex biological mixture. In this approach, a protein sample is enzymatically digested to produce peptides, which are then separated using capillary-scale columns packed with chromatographic media such as reversed phase, cation or anion exchange, or hydrophobic interaction resin (Wagner *et al.*, 2000). After ionization into an online tandem MS, the peptides are fragmented in the gas phase to generate uniquely informative product ion patterns. Computer-based interpretation of the resulting spectra using a database search algorithm can lead to the identification of the cognate parental proteins (Jalili & Dass, 2004). In the past several years, solution-based or gel-free technologies on the membrane proteome have gained popularity due to their excellent proteome sensitivity and rapid quantification efficiency (Hooker *et al.*, 2008; Poetsch & Wolters, 2008; Weiner & Li, 2008). For example, Slp, a lipoprotein attached to the OM that is associated with a starvation response, was identified through LC-MS analysis of cell extracts from an enteroinvasive *E. coli* strain (Spory *et al.*, 2002).

2D LC separations exploiting both the net solution charge state and the hydrophobicity of peptides can further boost the detection sensitivity of low-abundance membrane-bound proteins present in the cell-envelope compartments. Peptides are displaced from strong-cation-exchange resin using a salt step gradient and subsequently bind to a second reverse-phase media. Elution from the latter resin is accomplished using an organic (i.e. acetonitrile) gradient, with the peptides analyzed by standard MS/MS sequencing (Wu *et al.*, 2003). For example, a system comprised of two independent HPLC columns, one consisting of ion exchange preparative column and the other a reverse phase capillary column (Taoka *et al.*, 2004), was used to characterize several TIMPs (e.g. MrcB, MrcA, SecD and SecG) and TOMPs (e.g. ManX, NuoC) capable of forming stable oligomeric complexes in *E. coli* (Spelbrink *et al.*, 2005). Likewise, several periplasmic protein substrates (i.e. secreted) of the cytoplasmic chaperonin GroEL were detected using this screening technique (Chapman *et al.*, 2006). Conversely, the surface-oriented lysine residues of proteins associated with the IM can be differentially modified with dansyl chloride and then extracted after hydrolysis with chymotrypsin or proteinase K to shave off the exposed domains before selective enrichment and LC-MS identification of the labeled peptides (Cirulli *et al.*, 2007). Based on this approach, 29 putative TOMPs (e.g. YejO, FanD, YaeT and CssD), six lipoproteins (e.g. PgaB, NlpC and YdcL) and 43 TIMPs (e.g. YbbM, YiaH and YnfM) were identified. Organic compounds such as methanol have also been used to extract highly hydrophobic TIMPs from *E. coli*, outperforming solubilization with strong conventional detergents such as SDS when detected by 2D LC-MS/MS (Zhang *et al.*, 2007).

Quantitation and identification of membrane proteins can be achieved simultaneously by LC-MS using stable isotope-based mass tags running different samples in parallel (Thompson *et al.*, 2003). For example, about 5.5% of the cell-envelope subproteome was detected as differentially expressed using this approach in genetically perturbed *E. coli* cells under different exponential growth phase conditions with a confidence interval of >95% (Aggarwal *et al.*, 2005).

#### Green fluorescent protein (GFP) expression studies

Empirical criteria, such as the relative abundance of a membrane protein based on the expression levels of a GFP reporter fusion, can be used to examine the subcellular localization and dynamic range of the components of the cell-envelope. One of the most extensive GFP expression studies reported to date (Daley *et al.*, 2005) involved the C-terminal tagging of 714 putative TIMPs more than 100 amino acids long with at least two predicted α-helices (using TMHMM), with both alkaline phosphatase (PhoA) and GFP reporter fusions. Expression was quantified, and both localization and TIMP topology were established for *c*. 600 TIMPs. Further, Dr Hirotada Mori and colleagues (see Table S1 for the author's website) have performed an even more extensive GFP fusion localization study, wherein 90% of the ORFs in *E. coli* were tagged with GFP at the C-terminus, including 471 proteins with the GFP signal that was detected primarily as membrane proteins.

#### Summary of proteomic tools for deciphering protein subcellular localization

A number of proteomic methods for protein solubilization and detection of the membrane-related subproteome have been developed. Some of them have been adapted from both the water-soluble counterpart (as occurred analogously with the bioinformatic tools) and from the detection of complexes (the interactome), rather than isolated proteins (the proteome). What seems to be clear from our study is that more than one method is required to have greater efficiency in the detection of subcellular proteomes (see section ‘Integrating proteomic and bioinformatic tools to decipher the cell-envelope proteome’). For instance, 2D-PAGE analysis in yeast failed to detect many integral membrane proteins (Santoni *et al.*, 2000) requiring the use of specialized detergents and solubilization techniques. In the past several years, solution-based or gel-free technologies on the membrane proteome have gained popularity due to their excellent proteome sensitivity and rapid quantification efficiency (Hooker *et al.*, 2007; Poetsch & Wolters, 2008; Weiner & Li, 2008). Application of improvized protocols on the solubilization techniques with advance multidimensional LC separation should, in principle, allow us to scrutinize the *E. coli* membrane proteome with unparalleled sensitivity and accuracy.

### Integrating proteomic and bioinformatic tools to decipher the cell-envelope proteome

Contrary to bioinformatic studies, wherein each inputted protein (sequence) receives an independent score for its potential subcellular localization, proteomic analyses deciphering the subcellular localization of proteins contend with the fact that nearly all cellular processes involve physical associations between proteins, resulting in the formation of stable multimeric protein complexes as well as transient interactions (e.g. between a chaperone and its substrate) that are often viewed as potential ‘contaminants’ between compartments (Molloy *et al.*, 2000; Lopez-Campistrous *et al.*, 2005; Rey *et al.*, 2005b). It is plausible that a subset of such putative contaminants reflects *bona fide* physical PPIs or co-complex memberships. We therefore performed an ‘Agreement’-style analysis between the various proteomic and bioinformatic procedures and existing knowledge databases to determine the most frequently occurring potential contaminants.

In order to perform this analysis, we included four types of data sources. The first set comprises the subcellular localization assignments from five proteomic studies focused on specific compartments (Table 1). The second set consists of the 3503 predictions included in the ‘Majority Consensus’ of bioinformatic predictors of protein global subcellular localization (see section ‘Majority Consensus’ improves the prediction of global subcellular localization’ for details). The third set represents the reference databases ePSORTdb and TOPDB (Tusnády *et al.*, 2008). As described above, ePSORTdb is a widely used ‘gold standard’ database of proteins with well-documented subcellular localizations, while TOPDB is a database of experimentally derived TIMPs and TOMPs topologies. The fourth set includes other popular knowledge databases, such as Uniprot (Boeckmann *et al.*, 2003), EcoCyc (Karp *et al.*, 2007), CCDB (Sundararaj *et al.*, 2004), the Riley *et al.* (2006) annotation snapshot of the *E. coli* proteome and MultiFun (Serres *et al.*, 2004).

#### Major sources of common hits between proteomic and bioinformatic studies

**Fig. 4:**
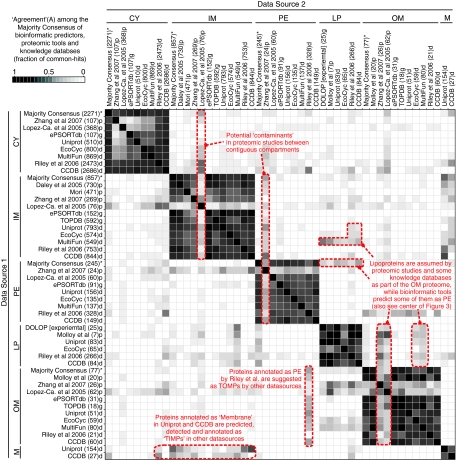
‘Agreement’ analysis between pairs of proteomic, bioinformatic tools and knowledge databases predicting or describing the *E. coli* cell-envelope-related proteome. Bioinformatic methods are represented by the ‘Majority Consensus’ of predictors of global subcellular localization (^*^). Proteomic studies are denoted by ‘p’, gold standard reference databases of protein subcellular localization are denoted by ‘g’ and other databases by ‘d’. Each square in the matrix represents the number of proteins predicted or described to be located in a given compartment by any two data sources. The darker the square intersecting any two data sources (D1 and D2), the higher the ‘Agreement’ between them (see section ‘Statistical parameters to evaluate the performance of predictors of subcellular localization’ for details). Predictions or descriptions of D1 are plotted on the *x*-axis, while predictions or descriptions of D2 are plotted on the *y*-axis. The number of predicted proteins for each subcellular location is shown in parentheses. Major discrepancies between datasources are highlighted in red frames. The list of cell-envelope proteins according to different proteomic methods is shown in Table S1. Subcellular localization acronyms are described as in Figs 1 and 3.

Our ‘Agreement’ analysis showed that the study of Daley *et al.* (2005) using GFP and PhoA fusions to establish protein subcellular localization ranks as the highest (*A*=0.97) in terms of common hits with the bioinformatic tools (represented by the ‘Majority Consensus’) (Fig. 4). While this result is expected, given that the authors used TMHMM, a predictor of α-helices, to select potential TIMPs (see Green fluorescent protein (GFP) expression studies) before analysis, we note that the other more global study by Dr H. Mori and colleagues using GFP to determine protein subcellular localization (http://ecoli.naist.jp/GFP/gfp_top.jsp) also ranked highly in terms of ‘Agreement’ (*A*=0.84), despite the fact that no bioinformatic prefiltering was seemingly used. Of the 471 ‘Membrane’ proteins (without specified status as TIMPs or TOMPs) analyzed by GFP in this study, we found that 395 of them (83%) are likely TIMPs based on the ‘Majority Consensus’, another 46 (10%) are predicted as cytoplasmic (although 10 contain one or two putative α-helices), one is predicted as OM and other as EC while the remaining 28 (5%) had no prediction, although Phobius predicted α-helices in 14, including the poorly characterized proteins HemY and HemX suggested to be a uroporphyrinogen III methylase (Sasarman *et al.*, 1988). This overlap of common hits between the GFP studies, bioinformatic predictors and knowledge databases provides strong evidence for both novel and corroborative TIMP annotations (see Table S1 for a detailed list), suggesting that an integrative approach can achieve high precision for detecting TIMPs (i.e. 80–90%).

In second place in terms of ‘Agreement’ with the bioinformatic tools (*A*=0.95) was the study of Molloy *et al.* (2000), using 2D-PAGE and MALDI-TOF to discover putative OM proteins (20 TOMPs and seven lipoproteins). Similarly, the average ‘Agreement’ of this study with knowledge databases was *A*=0.89. In contrast, we found a strikingly low overlap (*A*=0.35) between this study and the one of Zhang *et al.* (2007), who used SDS and methanol to solubilize TIMPs, followed by 2D LC-MS/MS for protein detection. Of the seven TOMPs detected as likely contaminants in the extracted IM fraction, one (Tsx) was reported as a TIMP (Zhang *et al.*, 2007). Nonetheless, Tsx is currently annotated in all analyzed databases as a TOMP, coinciding with the ‘Majority Consensus’ and the eight putative β-barrel strands detected.

The study of Zhang *et al.* (2007) was reported to have a precision of 40% (using SDS) and 44% (using methanol) for solubilizing and detecting integral membrane proteins. According to the ‘Majority Consensus’ and/or Phobius, we were able to predict α-helices for 207 out of 269 (77%) proteins reported as TIMPs by Zhang and colleagues, providing a *bona fide* core IM proteome. The authors used a composite of CCDB and GRAVY, a hydropathicity indicator based on the Kyte Doolitle index (see Bioinformatic approaches for investigating the cell-envelope proteome), to compare their experimental results with theoretical predictions, and inferred that at least 24 other proteins from the IM extracts were likely to be periplasmic contaminants. We noted, however, that 10 out of these 24 proteins are actually predicted as TIMPs by diverse global and feature-specific predictors (Fig. 4). For example, DipZ, MraY and YjeP have more than nine predicted α-helices, implying that a significant fraction of these potential periplasmic contaminants may in fact be *bona fide* TIMPs missed by GRAVY. Taking this and the observations on Tsx into account, the precision of the Zhang and colleagues method to solubilize and detect TIMPs consequently increased by *c*. 4% (see Table S1 for listing).

The study of Lopez-Campistrous *et al.*, (2005) showed a maximum ‘Agreement’ with the predictions of the water-soluble proteins, namely cytoplasmic (*A*=0.87) and periplasmic (*A*=0.78). This agrees with the fact that only 10 out of 368 (*c*. 2%) cytoplasmic proteins reported in this study have α-helices or β-strands determinants, strongly suggesting that the remaining 358 are *bona fide* cytoplasmic components (see Table S1 for the list). Similarly, 57 out of the 60 proteins suggested as periplasmic by Lopez-Campistrous *et al.* (2005) lack any predicted α-helix or β-strand. In contrast, the ‘Agreement’ between this proteomic study and both the ‘Majority Consensus’ (*A*=0.21) and the knowledge databases (average *A*=0.32) is low for the OM-associated proteome and even lower for the IM counterpart (*A*=0.18 for each datasource). This suggests that while the 2D-PAGE strategy used in this particular proteomic study was able to detect proteins from the four analyzed compartments (cytoplasm, IM, periplasm and OM), as elaborated below, the membrane-related proteomic fractions show a marked degree of contamination from the neighboring compartments.

#### Major sources of disagreement between proteomic and bioinformatic studies

Most of the outliers in the ‘Agreement’ analysis between the proteomic, bioinformatic and knowledgebases reside between neighboring compartments (represented by gray-to-black lines across different compartments in Fig. 4). For instance, 38 out of 78 proteins reported by Lopez-Campistrous *et al.* (2005), as purified from the IM, are suggested as cytoplasmic proteins by both the ‘Majority Consensus’ and several curated databases (Fig. 4). Furthermore, we were able to find α-helices in only 16 of these 78 proteins, including 12 with a single α-helix. This implies that at least half are cytoplasmic contaminants, while another four are both predicted as TOMPs in the ‘Majority Consensus’ and show more than 10 β-strands, strongly suggesting that they are also contaminants (see Table S1 for the list).

Notably, this same subset of 78 proteins had the lowest overall ‘Agreement’ (Fig. 4), representing a challenge for experimental biologists and bioinformaticians alike. In 2005, Lopez-Campistrous and colleagues reported that SWISS-PROT had most annotation as cytoplasmic, but a recent version of this database (v55.5) refers to 26 of them as IM, with the others currently having no curated subcellular annotation. Unfortunately, SWISS-PROT does not necessarily provide a source or means for revising misleading localizations. We were able to detect α-helices in only 11 of the 26 proteins referred as TIMPs by SWISS-PROT (see Table S1 for the list). The remaining 15 proteins seem to be annotated as ‘peripheral’ IM proteins, which do not span the IM *per se*– explaining the absence of TIMP α-helices – which associate with the IM via co-complex partner integral membrane subunits. This includes the cytoplasmic α, β, γ and ɛ subunits of the F(1)F(0) ATPase complex, which are bound to the IM via other subunits. Nonetheless, all the F(1)F(0) ATPase complex members are commonly annotated as ‘membrane bound’, which produces confusion by lexical-based predictors of subcellular localization. These observations illustrate the evolution of biological databases and bioinformatic predictors, and the need for continuous feedback with experimental biologists.

We also noted that 117 out of the 155 (75%) proteins with partial annotations referred to as ‘Membrane protein’ in UniProt (Boeckmann *et al.*, 2003) are currently annotated or predicted as IM in many other databases (Fig. 4), including 113 where α-helices were clearly predicted, providing a logical basis for a revised curation as TIMPs (see Table S1 for the list).

Other common sources of disagreement between bioinformatic and proteomic tools are the lipoproteins, which are commonly considered part of either the IM or the OM proteomes by experimentalists (Molloy *et al.*, 2000; Weiner & Li, 2008). A problem with this nomenclature is that predictors of global subcellular localization do not contemplate lipoproteins as a group. In fact, 40 out of the 93 (43%) lipoproteins of *E. coli* in the database DOLOP (known and predicted) do not have an assignment in the ‘Majority Consensus’, while another 24 (25%) are suggested as periplasmic, 11 each as OM and IM, six as cytoplasmic and one predicted as extracellular. In addition, 31 proteins tentatively annotated as periplasmic by Riley *et al.* (2006) are reported as OM in several other databases (Fig. 4, lower center). This suggests that regardless of semantic issues, the prediction and classification of lipoproteins represents a challenge for both experimentalists and bioinformaticians alike.

The 3D crystal structure of the lipoprotein Wza (Dong *et al.*, 2006), a translocon of capsular polysaccharides attached to the OM, is particularly useful to consider our claim: only 19 out of the 379 (5%) residues forming the sequence of Wza are actually embedded into the OM (where they surprisingly form an α-helix), while the bulk (*c*. 95%) of its structure is present in the periplasm, where it interacts with the rest of the polysaccharide translocon and the cell wall. Accordingly, the two SVM-based predictors of protein subcellular localization, based on protein amino acid composition (CELLO II and P-CLASSIFIER), predict Wza as a periplasmic protein, while the other predictors suggest that this protein resides in the OM (based on homology and previous annotations). This illustrates one of the potential sources of disagreement between proteomic and bioinformatic methods, but also among bioinformatic methods themselves.

#### Summary of the integration of proteomic and bioinformatic tools

Our ‘Performance’ and ‘Agreement’ analyses showed that both bioinformatic and proteomic tools display a high accuracy to determine the subproteome associated with specific compartments, for example the cytoplasm and the IM, while others such as the extracellular space, the OM and the classification of lipoproteins still represent a major challenge for both fields.

From our perspective, two of the most important challenges for forthcoming bioinformatic and proteomic assessments of protein subcellular localization include: (i) the prediction of subcellular localization of protein structural domains in addition to the global prediction schemes. This implies the integration of sequence and 3D-structure-based strategies for the detection of protein domain features that underlie the subcellular localization of protein regions. The hybrid nature of lipoproteins, such as Wza, partially membrane-embedded, partially water-soluble, occupies the tip of this fascinating challenge; and (ii) the detection and control of PPIs on the predictive power of protein subcellular localization, which, as described above, can represent a possible source of cross-contaminantion between contiguous compartments. This issue applies not only to proteomic studies but also to bioinformatic predictors, for instance to those based on text mining (e.g. the case of the ‘membrane-bound’ ATPase subunits). In the following section, we provide a summary of the most commonly used proteomic tools in an attempt to investigate the extent of PPIs between the components of various cell-envelope compartments.

## Advances in the proteomic approaches for elucidating the *E. coli* cell-envelope PPIs and protein complexes

### Low-throughput assays for detection of PPIs and protein complex co-membership interactions

A key feature of all biological systems is the tendency of proteins with related functions to associate physically via specific PPIs to form macromolecular complexes that work as molecular ‘machines’. The membrane-associated flagella, proton-motive ATP synthase and Type III secretion apparatus are extreme examples of such assemblies, but many other smaller multiprotein complexes are known or predicted to be associated with the cell-envelope, where they mediate diverse metabolic, signaling and transport activities within and between subcellular compartments.

In order to have an estimate of the current knowledge of protein complexes and PPIs occurring at the cell-envelope, we collected high-confidence PPIs deposited in three public databases, namely DIP (Salwinski *et al.*, 2004), BIND (Bader *et al.*, 2003) and IntAct (Hermjakob *et al.*, 2004) (Table 3). After excluding interactions from high-throughput assays (Butland *et al.*, 2005; Arifuzzaman *et al.*, 2006), which are treated in the next section, we refer to this collection of PPIs as the ‘PPI_lt’ network. Additionally, we collected heteromeric protein complexes described in EcoCyc (Karp *et al.*, 2007) and TCDB (Saier *et al.*, 2006) (Table 3 and Table S1); in this case, interactions should be considered as co-complex memberships (PCCMs), rather than direct physical PPIs.

**Table 3:** Data sources of experimental and bioinformatic PPI and protein functional interactions

Source	Data provided in each study	Reference
PPI and protein complexes		
DIP	Manually and automatically curated PPI	Hermjakob *et al.* (2004)
IntAct	Manually, automatically curated, and directly submitted biomolecular interactions	Xenarios *et al.* (2000)
BIND	Manually, automatically curated, and directly submitted biomolecular interactions, protein complexes and pathway information	Bader *et al.* (2003)
Butland *et al.*	A high-throughput PPI study	Butland *et al.* (2005)
TCDB	Manually curated transporter complexes classified functionally and evolutionarily	Saier *et al.* (2006)
Protein functional interactions		
Najafabadi & Salavati	Sequence-based prediction of protein functional interactions by means of codon usage	Najafabadi & Salavati (2008)
STRING	Known and predicted PPI and protein functional interactions derived from bioinformatic and experimental resources	von Mering *et al.* (2007)
NEBULON	Protein functional interactions predicted from operon predictions and rearrangements	Janga *et al.* (2005)

**Fig. 5:**
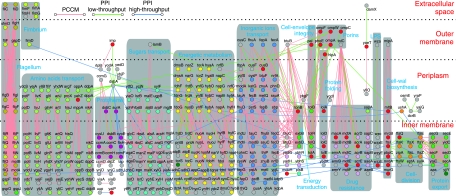
A census of the cell-envelope-related PPIs and protein complexes in knowledge databases. PPIs contained in the DIP, BIND and IntAct databases were filtered to obtain interactions derived from low-throughput (PPI_lt) and high-throughput (PPI_ht) experiments. Protein complex co-memberships (PCCM) annotated in the databases EcoCyc and TCDB are shown as edges connecting all-against-all proteins (nodes) forming a complex. Only interactions between proteins predicted as cell-envelope related according to the ‘Majority Consensus’ of predictors of global subcellular localization are shown. Node colors denote COG functional assignments, with the exception of grey nodes, where the poorly characterized proteins were assigned to categories ‘R and S, denoting proteins of no COG functional assignment. Proteins with grey nodes, depicted by blue labels, correspond to MultiFun functional assignments. Proteins depicted in red nodes were categorized under cell-envelope and OM biogenesis based on the COG functional assignment.

The union of the PPI_lt and PCCM networks (called ‘PPI_lt_U_PCCM’) reveals an extensively cross-connected graph (Fig. 5) dominated by interactions between components of diverse metabolite and drug transporters. Other notable interactions represented in this graph involve components of the flagellum and fimbriae, chaperones and protein-translocases and proteins involved in cell division. Importantly, the closer two compartments are to each other, the larger the number of PPIs between their respective protein components (inter-compartment interactions). For instance, considering the assignments in the ‘Majority Consensus’ of bioinformatic predictors of protein subcellular localization (see section ‘Majority Consensus’ improves the prediction of global subcellular localization’), we collected: 668 interactions (94 PPIs and 574 PCCMs) between pairs of TIMPs, 277 other interactions (69 PPIs and 208 PCCMs) between pairs of proteins, in which one component is a TIMP and the other is a cytoplasmic protein, and 250 interactions (17 PPIs and 233 PCCMs) between TIMPs and periplasmic proteins. This implies that in the current PPI_lt_U_PCCM network, for each 10 intracompartment interaction there are about four intercompartment links (e.g. IM vs. either the periplasm or the cytoplasm). In contrast, we found only 84 interactions (six PPIs and 78 PCCMs) between TIMPs and TOMPs (ratio 10 : 1.2). By generating a set of 1000 null models of the whole PPI_lt_U_PCCM network, in which all the interactions were randomly rewired but each node preserved exactly the same degree of connectivity (Maslov & Sneppen, 2002), we determined that inter-compartment interactions occur far more frequently than expected by chance (*P*<0.001) between proteins from adjacent neighbor compartments. This coincides with the results of the ‘Agreement’ analysis of proteomic tools (see section ‘Integrating proteomic and bioinformatic tools to decipher the cell-envelope proteome’), showing that potential contaminants in solubilization and detection of proteins tend to occur more between adjacent neighbor compartments (e.g. IM vs. periplasm, or IM vs. cytoplasm) than between more distant compartments (e.g. IM vs. OM).

We consider these results important based on the fact that some crossvalidation procedures applied to filter noise in high-throughput protein interaction studies from both proteomic (Shoemaker & Panchenko, 2007a) and bioinformatic inferences (Yellaboina *et al.*, 2007), assume that proteins in different compartments do not physically interact, but apparently that is not the case for neighboring compartments. Thus, we suggest that in addition to other PPIs benchmarking criteria such as gene coexpression (Shoemaker & Panchenko, 2007b), the construction of suitable negative gold standard reference data sets (proteins intended to not interact) makes appropriate use of proteins from subcellular locations separated by at least one another distinct compartment (e.g. cytoplasmic vs. periplasmic, or TIMPs vs. TOMPs).

### High-throughput protein complex co-membership detection by affinity co-purification

The subunits of solubilized but otherwise stable membrane multiprotein complexes tend to co-fractionate by density gradient centrifugation and exhibit differential retention on an ion exchange surface under native conditions (Hooker *et al.*, 2007). For instance, interactions between various TIMP and TOMP components of the tri-partite multidrug efflux pump, AcrAB-TolC, in *E. coli* were analyzed by this principle (Tikhonova & Zgurskaya, 2004). The relatively limited resolution and dynamic range of such procedures, however, has so far limited its applicability as a comprehensive screening approach.

In contrast, affinity co-purification approaches based on the use of specific epitope tags often allow for high-resolution isolation of protein complexes. For example, the classic Tandem Affinity Purification or TAP tag is a small specific polypeptidic sequence that is introduced in-frame into the C-terminus of a desired protein (bait). Because the sequence of the TAP tag can be recognized specifically by some proteins (e.g. antibodies and proteases) attached to an affinity column, the bait can be attached to the column via the TAP tag. Then, a ‘pull-down’ assay, which consists of passing a cellular extract over an affinity column, allows the selective retention of stable complexes based on co-purification of ‘prey’ proteins through their association with corresponding ‘bait’ (co-complex memberships). The protein complexes can be further detected by the fingerprint of their protein sequences by MALDI-TOF/MS or LC-MS/MS (Shoemaker & Panchenko, 2007a).

In *E. coli*, two large-scale ‘pull-down’ studies of the soluble proteome have been reported to date. In the first study (Butland *et al.*, 2005), the TAP method originally developed for yeast (Rigaut *et al.*, 1999) was modified to include a Sequential Peptide Affinity (SPA) dual tagging system (Zeghouf *et al.*, 2004). The SPA consists of a calmodulin-binding peptide, followed by the recognition site for the highly specific tobacco etch virus protease and three copies of a FLAG epitope integrated in-frame with the C-terminus of the target bait gene (Zeghouf *et al.*, 2004). The SPA tag confers sufficient affinity for calmodulin and M2 anti-FLAG affinity beads to enable successful recovery of low-abundance complexes from medium-scale cultures (typically 2–4 L of rich medium). Complementary MS procedures involving peptide mass fingerprinting by SDS-PAGE fractionation, followed by MALDI-TOF and shotgun peptide sequencing using gel-free LC MS/MS-based procedures, were then used to identify the interacting proteins with high sensitivity (i.e. low nanogram silver-stained limits). These procedures have the advantage of identifying endogenous native complexes as they exist *in vivo* (because the tagged protein is not overproduced). In the second case (Arifuzzaman *et al.*, 2006), hexahistidine-tagged baits were overexpressed as a means of isolating interactors before detection by MALDI-TOF MS.

**Fig. 6:**
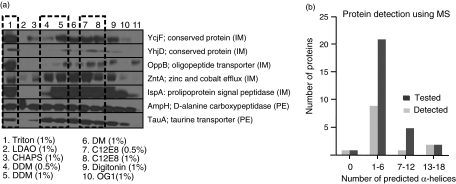
Selection of cell-envelope candidates for affinity tagging and purification using bioinformatic and proteomic data sources. (a) Western blotting of *E. coli* SPA-tagged TIMP and periplasmic proteins solubilized with eight different detergents, detected for the presence of the SPA-tag using an anti-FLAG antibody. The concentration of detergent used in the purification is shown in parentheses. The three detergents most effectively solubilizing the membrane proteins are indicated in a rectangular box with broken lines. The set of 34 candidates comprising of TIMP and periplasmic proteins was selected according to the predicted number of transmembrane α-helices and signal peptides, respectively, based on Phobius predictions (see Table S1 for the list). (b) SPA-purified *E. coli* membrane protein baits identified by mass spectrometry. The bar graph shows the recovery and detection coverage for affinity-tagged and -purified *E. coli* TIMP baits spanning both single membrane and polytopic (>10-TMH) transmembrane helices identified by MS. DM, *n*-dodecyl-β-d-maltoside. The acronyms of the other chemicals are described in the text.

In a recent pilot study by our group, we examined the solubilization efficiencies of eight different detergents selected for optimization of a representative set of SPA-tagged membrane proteins in a purification procedure compatible with the basic tandem purification procedure based on protocols cited in the literature (Kashino, 2003;Dobrovetsky *et al.*, 2005; Weiner & Li, 2008). Three detergents [1% DDM, 1% C12E8 (octaethylene glycol dodecyl ether) and 1% Triton X-100] were deemed to be quite effective, at least for bait extraction, as determined by Western blotting using an anti-FLAG antibody that detects the SPA tag (Fig. 6a). We next investigated how well these same three detergents performed in complete large-scale tandem purifications of 34 selected SPA-tagged *E. coli* cell-envelope proteins (see Table S1 for link). Despite the diversity in bait molecular size, function, predicted expression (using the Codon Adaptation Index) and number of predicted transmembrane α-helices, we were able to identify the bait and at least one putative co-complex partner for 17 of these baits (*c*. 50%). Both MALDI-TOF-MS and LC-MS/MS procedures were used, as from our experience one or the other technique occasionally misses certain proteins. We were able to detect 25 TIMPs that consisted of up to 12 predicted α-helices (Fig. 6b). Our sensitivity for peptide detection is typically better than 20 fmol, and the background is usually restricted to trace levels of ribosomal proteins, chaperones and a few other high-abundance common contaminants, as assessed by parallel purifications from untagged *E. coli* strains. On the other hand, we observed that our success rate in recovering or detecting the TIMPs with >10 predicted α-helices was reduced, suggesting that different detergents may be required to solubilize such proteins.

These preliminary results indicate that bacterial membrane protein complexes can potentially be systematically purified and characterized in the presence of optimally chosen nonionic detergents. In combination with highly sensitive MS, SPA-tag-based purification procedure should enable efficient detection of low-abundance *E. coli* membrane multiprotein complexes. Nevertheless, the quality of tentative physical interactors needs to be carefully validated, including verification by reciprocal tagging and purification, benchmarking with manually curated PPI databases such as DIP, BIND and IntAct, correlation with gene coexpression, elevated cooccurrence of orthologs in other species and other evidences of functional relatedness.

Despite the proven advantages in elucidating a large number of protein complex comembership networks using a dual-tagging approach, there are several inherent downsides with such a proteomic approach. The method involves labor-intensive tagging of each bait protein, requiring confirmation of tagged proteins with Western blotting, and subsequent large-scale purification of confirmed baits (Hooker *et al.*, 2007). Additionally, the purification of membrane proteins through this approach is much more complicated due to the inherent difficulty to solubilize membrane proteins without disrupting complex interactions, while the detergent has to be removed from the digested protein sample before LC-MS analysis because it can potentially interfere with peptide detection.

### Other proteomic and genetic approaches to decipher the cell-envelope proteome interactions

#### Protein co-IP

Historically, co-IP has been a handy method for verifying putative PPIs. This approach depends on the availability of specific antibodies or related capture agents to isolate a solubilized target protein antigen of interest and any interacting partners present within a sample. The complex is then typically detected by Western blotting using a second antibody targeted against one of the bound interacting proteins. For example, co-IP has been used to confirm the association of the two components of the twin-arginine (TatA and TatB) translocase complex in *E. coli* (Bolhuis *et al.*, 2000). Obvious limits reflect the difficulty in scaling up reagent production to investigate an entire proteome.

#### Bacterial two-hybrid system

Two-hybrid screening systems have been shown to be promising in elucidating binary interactions among membrane proteins as this technology avoids the need to co-purify intact complexes. For example, this method has been used to detect pair-wise interactions between membrane proteins involved in cell division (Karimova *et al.*, 2005) and in the protein folding quality control mechanism of the secretion Tat pathway (Strauch & Georgiou, 2007). Large-scale PPI screens have also revealed interactions between novel and known components of two models of bacterial motility, namely *Campylobacter jejuni* and *Helicobacter pylori* (Rajagopala *et al.*, 2007). The Keio *E. coli* strain collection (Baba *et al.*, 2006) of single gene deletion mutants was subseqeuntly used to confirm novel components of the bacterial motility network by phenotypic analysis (Rajagopala *et al.*, 2007).

#### Fluorescence resonance energy transfer (FRET)

FRET has been useful for monitoring membrane protein interactions in live cells. The technique is based on the energy transfer between two closely positioned fluorescent proteins that are fused to two interacting protein partners (Link *et al.*, 2007). Recruitment of two-component signaling systems of chemotactic response receptors in *E. coli*, including IM localized protein kinases, has been mapped using this technique (Vaknin & Berg, 2004), which has been suggested as a promising routine tool for determining transmembrane protein interactions (Hooker *et al.*, 2007). Conversely, the molecular mass of a membrane protein complex can also be measured through the fluctuations in the fluorescence intensity derived from an illuminated region or through the translational diffusion coefficient during fluorescence correlation spectroscopy (FCS). For example, aggregation of the MinD protein on the IM (Meacci *et al.*, 2006) and the tumbling rate of the *E. coli* flagellum (Cluzel *et al.*, 2000) were measured using this technique. Likewise, proteins labeled with two different fluorescent dyes can be concurrently excited by two different lasers and monitored by fluorescence cross-correlation spectroscopy (FCCS) (Schwille *et al.*, 1997). The fluorescent signals are then split by a photon burst, which further enables monitoring the fluorescence of the dyes individually. The cross-correlation function is subsequently determined by measuring the amplitude of the product concentration of the diffusing particles carrying both dyes. This technique can potentially determine the stoichiometry of protein interactions by means of the diffusion characteristics. This method may be more appropriate for membrane proteins than FCS due to the limited mobility of single membrane-bound ligands (Hooker *et al.*, 2007). For example, FCCS was used to measure the oligomeric state and stability of the mannitol transporter from *E. coli*, EnzymeII^mtl^, a member of the phosphoenolpyruvate-dependent phosphotransferase enzyme in the IM lipid bilayer (Veldhuis *et al.*, 2006).

#### SPR

The SPR method allows determination of the direct physical interactions of two purified proteins *in vitro* via changes in the light refractive index of one of the proteins that is tethered to a solid phase (Visudtiphole *et al.*, 2006; Hooker *et al.*, 2007). SPR has been used to monitor the assembly and dynamics of a signal transduction complex that controls chemotaxis in *E. coli*. Using this approach, a quaternary complex was shown to be formed between the response regulator CheY, the histidine protein kinase CheA, Tar (a TIMP chemoreceptor) and CheW (Schuster *et al.*, 1993).

#### Use of PPI and protein complex co-membership networks in drug target discovery

One of the major aims in Biomedical Sciences is the use of Systems Biology-based research for the discovery of potential novel drug targets, leading, for example, to the inhibition and/or the ablation of critical effector proteins of pathogens (Ivanov *et al.*, 2007). Diverse drugs are known to block or alter the biogenesis or the proper functions of essential pathways of the cell-envelope; for instance, the β-lactams (e.g. penicillin and ampicillin) and glycopeptides (e.g. vancomycin) inhibit formation of the cell wall, while polymyxin disrupts formation of the OM. Although the specific targets of certain antibiotics are not fully documented, the ability of others to alter specific pathways involves highly selective binding into specific protein pockets (e.g. enzyme-active sites) by mimicking naturally occurring substrates or ligands (Kuhn *et al.*, 2008a). The database STITCH (Kuhn *et al.*, 2008b) (a companion of the widely used Search Tool for the Retrieval of Interacting Genes/Proteins, STRING) provides known and predicted interactions between proteins and drugs using genomic context-based inferences and text mining protocols.

In the case of PPIs, small molecules termed ‘dimerizers’ can potentially induce physical interactions leading to altered cellular responses (Michnick, 2000; Archakov *et al.*, 2003), while others can prevent the formation of protein complexes (Cochran, 2000, 2001; Archakov *et al.*, 2003). In *E. coli*, the 3D crystal structure of ZipA (Mosyak *et al.*, 2000), a TIMP that plays an important role in the formation of the septal ring essential for cell division, has been solved in complex with a 17-residue peptide from FtsZ, another protein participating in cell division. A couple of small molecule inhibitors of the ZipA–FtsZ interaction have been developed to show that binding affinities displayed by the FtsZ peptide can potentially identify new drugs disrupting the PPI (Fry, 2006).

### Functional screens and genetic (epistatic) interaction surveys

It has been suggested that genes encoding highly connected proteins in PPI networks tend to be essential for survival (Jeong *et al.*, 2001). However, in *E. coli*, only 303 genes (7% of the entire genome) appear to be essential for survival under standard laboratory growth conditions (Baba *et al.*, 2006), suggesting some level of functional redundancy or buffering between the remaining pathway components. In some cases, simultaneous mutation or knock-out of different genes in parallel or converging pathways can result in a phenotype that is more striking than expected from the multiplicative effects of the single gene deletion defects alone. The extreme case of this synthetic effect is cell death or inviability (synthetic lethality). For example, the periplasmic chaperones Skp and DegP have been described as forming redundant pathways with SurA (Rizzitello *et al.*, 2001) as the simultaneous deletion of genes encoding these proteins results in a synthetic aggravating growth phenotype. Double mutants producing synthetic sick or lethal effects are commonly referred as ‘SSL’, whereas double mutants showing epistasis resulting in better growth are termed as alleviating interactions.

Technologies to perform systematic genome-wide surveys of genetic interactions (including SSL) were developed recently by our group (Butland *et al.*, 2008) and by Gross and colleagues (Typas *et al.*, 2008) to elucidate the global pathway architecture of *E. coli*. The strategy (called eSGA, ‘*E. coli* synthetic genetic array analysis’ by our group) is based on natural bacterial conjugation between High frequency of recombination (Hfr) query gene deletion mutant, which are crossed against a collection of 3850 single-gene deletion mutants (‘Keio collection’) covering all *E. coli* nonessential genes (Baba *et al.*, 2006). The relative fitness of the resulting double-mutant strains is measured based on the colony growth to determine SSL interactions. Through this approach, we have shown that the simultaneous deletion of genes participating in two alternative pathways (Isc and Suf), involved in the metabolism of Fe-S clusters, results in SSL double mutants (Butland *et al.*, 2008). Immunolocalization studies have shown that SufB and SufC proteins in Suf pathway bound to IM in *E. coli* (Rangachari *et al.*, 2002), coinciding with predictions from Proteome Analyst [see section ‘Predictors using lexical (keyword) annotations’], while components of the Isc pathway, such as IscS, appear to be important for the activity of both cytoplasmic and membrane-bound Fe-S enzymes (Schwartz *et al.*, 2000). Additionally, integrating genomic context-based inferences with PPIs and eSGA, we were able to determine that a putative TIMP of unknown function YfbJ participates in the metabolism of lipid A and other sugars necessary for the biogenesis of the OM (P. Hu *et al.*, unpublished data), coinciding with a recent work suggesting that YfbJ serves as a transporter for Lipid A precursors (Yan *et al.*, 2007).

The analogous GIANT-coli strategy reported by Gross and collaborators (Typas *et al.*, 2008) was used to investigate interactions among a set of 12 genes involved in the biogenesis of the cell-envelope (12 × 12 crosses). Their results highlight nine SSL and four alleviating interactions in rich media, some of which were more pronounced in minimal medium. For instance, the double mutant Δ*ompA-*Δ*pal* shows a sick phenotype in rich media, while it showed lethality in minimal media.

Taken together, in principle, large-scale genetic interaction screens based on these strategies should allow large-scale mapping of *E. coli* genetic interaction networks across the entire cell-envelope, defining the overall functional architecture of interlinked membrane PPIs and protein complexes, and provide insights into the mechanistic basis behind assembly of the membrane bilayers and the cell wall.

### Other phenotypic assays involving gene deletion mutants

In addition to detecting growth fitness defects, single- and double-mutant strain collections can be used to uncover other types of phenotypic alterations. For example, FimH, a protein generally associated with type I pili formation, was demonstrated to be required for initial surface attachment during biofilm formation (Pratt & Kolter, 1998). Analogous large-scale genetic screens have been used to determine several novel components needed for bacterial motility. In the first study (Rajagopala *et al.*, 2007), bioinformatic tools were combined with swarming motility assays to identify candidate genes involved in the bacterial motility of five bacterial species, including *E. coli*. The predictions were verified in strains present in the Keio collection of *E. coli* single-gene knock-out mutants, and a parallel *B. subtilis* mutant counterpart. Later, a whole-genome scan was conducted in *C. jejuni* and *H. pylori* using the bacterial two-hybrid system to determine a conserved network of proteins with 23 novel components involved in motility (Rajagopala *et al.*, 2007). In the second study (Girgis *et al.*, 2007), *E. coli* single and double mutants were used to map the genetic architecture behind bacterial motility. In this case, the strategy involves competitive selection and microarray-based genetic mapping of bacterial behaviors, revealing 36 novel components of the *E. coli* motility network and several epistatic interactions, including both SSL and alleviating phenotypes, most of which affect the production of lipopolysaccharide (Girgis *et al.*, 2007).

Although synthetic lethal interactions are assumed to involve two or more genes, the spectrum of synthetic relationships and genetic interactions encompasses effects dependent on environmental stresses or chemical perturbations, which mimic or aggravate deletion outcomes. In *E. coli* for example, a recent genomic-scale study (Tamae *et al.*, 2008) provided data on the phenotypic consequences of exposing single gene deletion mutants to one of seven different types of antibiotics, including ampicillin, an inhibitor of the bacterial cell-wall biogenesis. As expected, several of the most strongly sensitive mutants had defects in genes encoding penicillin-binding proteins, such as *mrcB* and *dacA*, which participates in the assembly of the cell-wall biogenesis.

As a complement to the proteomic methods to decipher physical and functional protein interactions, a number of bioinformatic methods have been developed. A detailed description of these methods is beyond the scope of this work; however, the reader is referred to some recent reviews on this topic (Sharan *et al.*, 2007; Shoemaker & Panchenko, 2007b). A list of databases providing diverse sources of known and predicted physical and functional interactions is provided in Table 3.

## Concluding remarks

In this review, we have examined various experimental and computational approaches to gain an insight into the architecture of the bacterial cell-envelope. From our point of view, none of these methods alone can fully elucidate the underlying mechanistic processes occurring in *E. coli*, or any other living system. Integration of data generated from various platforms should in principle be instrumental in developing improved inference procedures and in producing more reliable information on the broader implications of the cell-envelope-associated proteome, and in particular the *E. coli* membrane proteome biology. Proteomic and genetic approaches for deciphering the physical and functional architecture of the cell-envelope proteome can be significantly enhanced with the aid of bioinformatic tools and databases specialized in protein subcellular localization.

In our opinion, computational predictors of global subcellular localization and specific feature detectors need to be incorporated into routine experimental laboratory procedures, in a manner similar to how blast or multiple alignment programs are commonly used. Bioinformaticians in turn need to develop software that interfaces into experimental pipelines seamlessly. Overall, the availability of precomputed bacterial genome-scale predictions would be highly beneficial. On the other hand, while contemporary bioinformatic predictors of global subcellular localization are generally effective, in particular for determining the cytoplasmic and IM-related proteomes, deciphering the OM, periplasmic and extracellular-related subproteomes still represents a major challenge. Lipoprotein ‘flags’ are a desirable feature that, although provided by individual signal peptide detectors, is not incorporated by current predictors of global subcellular localization.

Overall, our analyses strongly suggest that integration of different methods results in more sensitive and precise predictions than those obtained by separate methods alone. The simple integrative ‘Majorty Consensus’ provided in this work suggests that more elaborate integration strategies, for example by weighting the votes from different predictors according to their observed performance for different subcellular compartments, should result in more highly accurate predictions. In this sense, the construction of meta-servers is imperative. We also noticed that an important number of putative contaminants in proteomic studies tend to come from contiguous compartments, reflecting in part genuine PPIs. This phenomenon needs to be taken into account when benchmarking bioinformatic predictors and proteomic studies of protein interactions.

## Statement

Reuse of this article is permitted in accordance with the Creative Commons Deed, Attribution 2.5, which does not permit commercial exploitation.

## Supporting Information

**Table S1.** Compilation and evaluation of subcellular localization predictions and annotations of the *E. coli* K-12 proteome.

**Table S2.** Compilation of bioinformatic predictions of subcellular localization for a set of 299 proteins from Gram-negative (gold standard) bacteria.

## References

[b1] Aggarwal K, Choe LH, Lee KH (2005). Quantitative analysis of protein expression using amine-specific isobaric tags in *Escherichia coli* cells expressing rhsA elements. Proteomics.

[b2] Alami M, Dalal K, Lelj-Garolla B, Sligar SG, Duong F (2007). Nanodiscs unravel the interaction between the SecYEG channel and its cytosolic partner SecA. EMBO J.

[b3] Alexander RP, Zhulin IB (2007). Evolutionary genomics reveals conserved structural determinants of signaling and adaptation in microbial chemoreceptors. P Natl Acad Sci USA.

[b4] Altschul SF, Madden TL, Schaffer AA, Zhang J, Zhang Z, Miller W, Lipman DJ (1997). Gapped BLAST and PSI-BLAST: a new generation of protein database search programs. Nucleic Acids Res.

[b5] Andrade MA, Brown NP, Leroy C (1999). Automated genome sequence analysis and annotation. Bioinformatics.

[b6] Arai M, Mitsuke H, Ikeda M, Xia JX, Kikuchi T, Satake M, Shimizu T (2004). ConPred II: a consensus prediction method for obtaining transmembrane topology models with high reliability. Nucleic Acids Res.

[b7] Archakov AI, Govorun VM, Dubanov AV, Ivanov YD, Veselovsky AV, Lewi P, Janssen P (2003). Protein–protein interactions as a target for drugs in proteomics. Proteomics.

[b8] Arifuzzaman M, Maeda M, Itoh A (2006). Large-scale identification of protein–protein interaction of *Escherichia coli* K-12. Genome Res.

[b9] Ashburner M, Ball CA, Blake JA (2000). Gene ontology: tool for the unification of biology. The gene ontology consortium. Nat Genet.

[b10] Baba T, Ara T, Hasegawa M, Takai Y, Okumura Y, Baba M, Datsenko KA, Tomita M, Wanner BL, Mori H (2006). Construction of *Escherichia coli* K-12 in-frame, single-gene knockout mutants: the Keio collection. Mol Syst Biol.

[b11] Babu MM, Priya ML, Selvan AT, Madera M, Gough J, Aravind L, Sankaran K (2006). A database of bacterial lipoproteins (DOLOP) with functional assignments to predicted lipoproteins. J Bacteriol.

[b12] Backert S, Meyer TF (2006). Type IV secretion systems and their effectors in bacterial pathogenesis. Curr Opin Microbiol.

[b13] Bader GD, Betel D, Hogue CW (2003). BIND: the biomolecular interaction network database. Nucleic Acids Res.

[b14] Bagos PG, Liakopoulos TD, Spyropoulos IC, Hamodrakas SJ (2004). PRED-TMBB: a web server for predicting the topology of beta-barrel outer membrane proteins. Nucleic Acids Res.

[b15] Bagos PG, Liakopoulos TD, Hamodrakas SJ (2005). Evaluation of methods for predicting the topology of beta-barrel outer membrane proteins and a consensus prediction method. BMC Bioinformatics.

[b16] Bayan N, Guilvout I, Pugsley AP (2006). Secretins take shape. Mol Microbiol.

[b17] Behrens S (2003). Periplasmic chaperones – preservers of subunit folding energy for organelle assembly. Cell.

[b18] Bendtsen JD, Nielsen H, von Heijne G, Brunak S (2004). Improved prediction of signal peptides: SignalP 3.0. J Mol Biol.

[b19] Bendtsen JD, Nielsen H, Widdick D, Palmer T, Brunak S (2005). Prediction of twin-arginine signal peptides. BMC Bioinformatics.

[b20] Berg HC (2003). The rotary motor of bacterial flagella. Annu Rev Biochem.

[b21] Bernsel A, Viklund H, Falk J, Lindahl E, von Heijne G, Elofsson A (2008). Prediction of membrane-protein topology from first principles. P Natl Acad Sci USA.

[b22] Berven FS, Flikka K, Jensen HB, Eidhammer I (2004). BOMP: a program to predict integral beta-barrel outer membrane proteins encoded within genomes of gram-negative bacteria. Nucleic Acids Res.

[b23] Bigelow HR, Petrey DS, Liu J, Przybylski D, Rost B (2004). Predicting transmembrane beta-barrels in proteomes. Nucleic Acids Res.

[b24] Boeckmann B, Bairoch A, Apweiler R (2003). The SWISS-PROT protein knowledgebase and its supplement TrEMBL in 2003. Nucleic Acids Res.

[b25] Bolhuis A, Bogsch EG, Robinson C (2000). Subunit interactions in the twin-arginine translocase complex of *Escherichia coli*. FEBS Lett.

[b26] Bos MP, Robert V, Tommassen J (2007). Biogenesis of the gram-negative bacterial outer membrane. Annu Rev Microbiol.

[b27] Braun M, Killmann H, Maier E, Benz R, Braun V (2002). Diffusion through channel derivatives of the *Escherichia coli* FhuA transport protein. Eur J Biochem.

[b28] Braun RJ, Kinkl N, Beer M, Ueffing M (2007). Two-dimensional electrophoresis of membrane proteins. Anal Bioanal Chem.

[b29] Brutinel ED, Yahr TL (2008). Control of gene expression by type III secretory activity. Curr Opin Microbiol.

[b30] Butland G, Peregrin-Alvarez JM, Li J (2005). Interaction network containing conserved and essential protein complexes in *Escherichia coli*. Nature.

[b31] Butland G, Babu M, Diaz-Mejia JJ (2008). eSGA: *E. coli* synthetic genetic array analysis. Nat Methods.

[b32] Cedano J, Aloy P, Perez-Pons JA, Querol E (1997). Relation between amino acid composition and cellular location of proteins. J Mol Biol.

[b33] Chapman E, Farr GW, Usaite R (2006). Global aggregation of newly translated proteins in an *Escherichia coli* strain deficient of the chaperonin GroEL. P Natl Acad Sci USA.

[b34] Chou KC, Cai YD (2003). A new hybrid approach to predict subcellular localization of proteins by incorporating gene ontology. Biochem Bioph Res Co.

[b35] Chou KC, Shen HB (2006). Large-scale predictions of gram-negative bacterial protein subcellular locations. J Proteome Res.

[b36] Cirulli C, Marino G, Amoresano A (2007). Membrane proteome in *Escherichia coli* probed by MS3 mass spectrometry: a preliminary report. Rapid Commun Mass Sp.

[b37] Claros MG, von Heijne G (1994). TopPred II: an improved software for membrane protein structure predictions. Comput Appl Biosci.

[b38] Cluzel P, Surette M, Leibler S (2000). An ultrasensitive bacterial motor revealed by monitoring signaling proteins in single cells. Science.

[b39] Cochran AG (2000). Antagonists of protein–protein interactions. Chem Biol.

[b40] Cochran AG (2001). Protein–protein interfaces: mimics and inhibitors. Curr Opin Chem Biol.

[b41] Collin S, Guilvout I, Chami M, Pugsley AP (2007). YaeT-independent multimerization and outer membrane association of secretin PulD. Mol Microbiol.

[b42] Daley DO, Rapp M, Granseth E, Melen K, Drew D, von Heijne G (2005). Global topology analysis of the *Escherichia coli* inner membrane proteome. Science.

[b43] Dobrovetsky E, Lu ML, Andorn-Broza R, Khutoreskaya G, Bray JE, Savchenko A, Arrowsmith CH, Edwards AM, Koth CM (2005). High-throughput production of prokaryotic membrane proteins. J Struct Funct Genom.

[b44] Doerrler WT (2006). Lipid trafficking to the outer membrane of gram-negative bacteria. Mol Microbiol.

[b45] Dong C, Beis K, Nesper J, Brunkan-Lamontagne AL, Clarke BR, Whitfield C, Naismith JH (2006). Wza the translocon for *E. coli* capsular polysaccharides defines a new class of membrane protein. Nature.

[b46] Driessen AJ, Nouwen N (2008). Protein translocation across the bacterial cytoplasmic membrane. Annu Rev Biochem.

[b47] Emanuelsson O, Brunak S, von Heijne G, Nielsen H (2007). Locating proteins in the cell using TargetP, SignalP and related tools. Nat Protoc.

[b48] Filloux A, Hachani A, Bleves S (2008). The bacterial type VI secretion machine: yet another player for protein transport across membranes. Microbiology.

[b49] Fountoulakis M, Gasser R (2003). Proteomic analysis of the cell envelope fraction of *Escherichia coli*. Amino Acids.

[b50] Fry DC (2006). Protein–protein interactions as targets for small molecule drug discovery. Biopolymers.

[b51] Galan JE, Wolf-Watz H (2006). Protein delivery into eukaryotic cells by type III secretion machines. Nature.

[b52] Gama-Castro S, Jimenez-Jacinto V, Peralta-Gil M (2008). RegulonDB (version 6.0): gene regulation model of *Escherichia coli* K-12 beyond transcription, active (experimental) annotated promoters and Textpresso navigation. Nucleic Acids Res.

[b53] Garcia DL, Dillard JP (2008). Mutations in ampG or ampD affect peptidoglycan fragment release from *Neisseria gonorrhoeae*. J Bacteriol.

[b54] Gardy JL, Brinkman FS (2006). Methods for predicting bacterial protein subcellular localization. Nat Rev Microbiol.

[b55] Gardy JL, Laird MR, Chen F, Rey S, Walsh CJ, Ester M, Brinkman FS (2005). PSORTb v.2.0: expanded prediction of bacterial protein subcellular localization and insights gained from comparative proteome analysis. Bioinformatics.

[b56] Garrow AG, Agnew A, Westhead DR (2005). TMB-Hunt: an amino acid composition based method to screen proteomes for beta-barrel transmembrane proteins. BMC Bioinformatics.

[b57] Gerding MA, Ogata Y, Pecora ND, Niki H, de Boer PA (2007). The trans-envelope Tol-Pal complex is part of the cell division machinery and required for proper outer-membrane invagination during cell constriction in *E. coli*. Mol Microbiol.

[b58] Girgis HS, Liu Y, Ryu WS, Tavazoie S (2007). A comprehensive genetic characterization of bacterial motility. PLoS Genet.

[b59] Gromiha MM, Suwa M (2005). A simple statistical method for discriminating outer membrane proteins with better accuracy. Bioinformatics.

[b60] Gromiha MM, Ahmad S, Suwa M (2005). TMBETA-NET: discrimination and prediction of membrane spanning beta-strands in outer membrane proteins. Nucleic Acids Res.

[b61] Gromiha MM, Yabuki Y, Kundu S, Suharnan S, Suwa M (2007a). TMBETA-GENOME: database for annotated beta-barrel membrane proteins in genomic sequences. Nucleic Acids Res.

[b62] Gromiha MM, Yabuki Y, Suwa M (2007b). TMB finding pipeline: novel approach for detecting beta-barrel membrane proteins in genomic sequences. J Chem Inf Model.

[b63] Han MJ, Lee SY (2006). The *Escherichia coli* proteome: past, present, and future prospects. Microbiol Mol Biol Rev.

[b64] Haselbeck R, Wall D, Jiang B, Ketela T, Zyskind J, Bussey H, Foulkes JG, Roemer T (2002). Comprehensive essential gene identification as a platform for novel anti-infective drug discovery. Curr Pharm Design.

[b65] Hedman M, Deloof H, Von Heijne G, Elofsson A (2002). Improved detection of homologous membrane proteins by inclusion of information from topology predictions. Protein Sci.

[b66] Hermjakob H, Montecchi-Palazzi L, Lewington C (2004). IntAct: an open source molecular interaction database. Nucleic Acids Res.

[b67] Hernandez-Montes G, Diaz-Mejia JJ, Perez-Rueda E, Segovia L (2008). The hidden universal distribution of amino acid biosynthetic networks: a genomic perspective on their origins and evolution. Genome Biol.

[b68] Hirashima A, Wang S, Inouye M (1974). Cell-free synthesis of a specific lipoprotein of the *Escherichia coli* outer membrane directed by purified messenger RNA. P Natl Acad Sci USA.

[b69] Holland IB, Schmitt L, Young J (2005). Type 1 protein secretion in bacteria, the ABC-transporter dependent pathway (review). Mol Membr Biol.

[b70] Holst O (2007). The structures of core regions from enterobacterial lipopolysaccharides – an update. FEMS Microbiol Lett.

[b71] Hooker BS, Bigelow DJ, Lin CT (2007). Methods for mapping of interaction networks involving membrane proteins. Biochem Bioph Res Co.

[b72] Hritonenko V, Stathopoulos C (2007). Omptin proteins: an expanding family of outer membrane proteases in gram-negative Enterobacteriaceae. Mol Membr Biol.

[b73] Ivanov AS, Gnedenko OV, Molnar AA, Mezentsev YV, Lisitsa AV, Archakov AI (2007). Protein–protein interactions as new targets for drug design: virtual and experimental approaches. J Bioinform Comput Biol.

[b74] Jalili PR, Dass C (2004). Proteome analysis in the bovine adrenal medulla using liquid chromatography with tandem mass spectrometry. Rapid Commun Mass Sp.

[b75] Janga SC, Collado-Vides J, Moreno-Hagelsieb G (2005). Nebulon: a system for the inference of functional relationships of gene products from the rearrangement of predicted operons. Nucleic Acids Res.

[b76] Jeong H, Mason SP, Barabasi AL, Oltvai ZN (2001). Lethality and centrality in protein networks. Nature.

[b77] Jones DT (2007). Improving the accuracy of transmembrane protein topology prediction using evolutionary information. Bioinformatics.

[b78] Juncker AS, Willenbrock H, Von Heijne G, Brunak S, Nielsen H, Krogh A (2003). Prediction of lipoprotein signal peptides in Gram-negative bacteria. Protein Sci.

[b79] Kall L, Krogh A, Sonnhammer EL (2004). A combined transmembrane topology and signal peptide prediction method. J Mol Biol.

[b80] Karimova G, Dautin N, Ladant D (2005). Interaction network among *Escherichia coli* membrane proteins involved in cell division as revealed by bacterial two-hybrid analysis. J Bacteriol.

[b81] Karp PD, Keseler IM, Shearer A (2007). Multidimensional annotation of the *Escherichia coli* K-12 genome. Nucleic Acids Res.

[b82] Kashino Y (2003). Separation methods in the analysis of protein membrane complexes. J Chromatogr B.

[b83] Kerrien S, Alam-Faruque Y, Aranda B (2007). IntAct – open source resource for molecular interaction data. Nucleic Acids Res.

[b84] Keseler IM, Collado-Vides J, Gama-Castro S, Ingraham J, Paley S, Paulsen IT, Peralta-Gil M, Karp PD (2005). EcoCyc: a comprehensive database resource for *Escherichia coli*. Nucleic Acids Res.

[b85] Kim S, Malinverni JC, Sliz P, Silhavy TJ, Harrison SC, Kahne D (2007). Structure and function of an essential component of the outer membrane protein assembly machine. Science.

[b86] Kleinschmidt JH (2003). Membrane protein folding on the example of outer membrane protein A of *Escherichia coli*. Cell Mol Life Sci.

[b87] Krause F (2006). Detection and analysis of protein–protein interactions in organellar and prokaryotic proteomes by native gel electrophoresis: (membrane) protein complexes and supercomplexes. Electrophoresis.

[b88] Krogh A, Larsson B, von Heijne G, Sonnhammer EL (2001). Predicting transmembrane protein topology with a hidden Markov model: application to complete genomes. J Mol Biol.

[b89] Krojer T, Sawa J, Schafer E, Saibil HR, Ehrmann M, Clausen T (2008). Structural basis for the regulated protease and chaperone function of DegP. Nature.

[b90] Kuhn M, Campillos M, Gonzalez P, Jensen LJ, Bork P (2008a). Large-scale prediction of drug-target relationships. FEBS Lett.

[b91] Kuhn M, von Mering C, Campillos M, Jensen LJ, Bork P (2008b). STITCH: interaction networks of chemicals and proteins. Nucleic Acids Res.

[b92] Kustos I, Kocsis B, Kilar F (2007). Bacterial outer membrane protein analysis by electrophoresis and microchip technology. Expert Rev Proteomics.

[b93] Kyte J, Doolittle RF (1982). A simple method for displaying the hydropathic character of a protein. J Mol Biol.

[b94] Lao DM, Arai M, Ikeda M, Shimizu T (2002). The presence of signal peptide significantly affects transmembrane topology prediction. Bioinformatics.

[b95] Lasserre JP, Beyne E, Pyndiah S, Lapaillerie D, Claverol S, Bonneu M (2006). A complexomic study of *Escherichia coli* using two-dimensional blue native/SDS polyacrylamide gel electrophoresis. Electrophoresis.

[b96] Lee PA, Tullman-Ercek D, Georgiou G (2006). The bacterial twin-arginine translocation pathway. Annu Rev Microbiol.

[b97] Link AJ, Jeong KJ, Georgiou G (2007). Beyond toothpicks: new methods for isolating mutant bacteria. Nat Rev Microbiol.

[b98] Lopez-Campistrous A, Semchuk P, Burke L, Palmer-Stone T, Brokx SJ, Broderick G, Bottorff D, Bolch S, Weiner JH, Ellison MJ (2005). Localization, annotation, and comparison of the *Escherichia coli* K-12 proteome under two states of growth. Mol Cell Proteomics.

[b99] Lu P, Szafron D, Greiner R, Wishart DS, Fyshe A, Pearcy B, Poulin B, Eisner R, Ngo D, Lamb N (2005). PA-GOSUB: a searchable database of model organism protein sequences with their predicted gene ontology molecular function and subcellular localization. Nucleic Acids Res.

[b100] Lu Z, Szafron D, Greiner R, Lu P, Wishart DS, Poulin B, Anvik J, Macdonell C, Eisner R (2004). Predicting subcellular localization of proteins using machine-learned classifiers. Bioinformatics.

[b101] Madan Babu M, Sankaran K (2002). DOLOP – database of bacterial lipoproteins. Bioinformatics.

[b102] Maslov S, Sneppen K (2002). Specificity and stability in topology of protein networks. Science.

[b103] Meacci G, Ries J, Fischer-Friedrich E, Kahya N, Schwille P, Kruse K (2006). Mobility of Min-proteins in *Escherichia coli* measured by fluorescence correlation spectroscopy. Phys Biol.

[b104] Michnick SW (2000). Chemical biology beyond binary codes. Chem Biol.

[b105] Molloy MP, Herbert BR, Williams KL, Gooley AA (1999). Extraction of *Escherichia coli* proteins with organic solvents prior to two-dimensional electrophoresis. Electrophoresis.

[b106] Molloy MP, Herbert BR, Slade MB, Rabilloud T, Nouwens AS, Williams KL, Gooley AA (2000). Proteomic analysis of the *Escherichia coli* outer membrane. Eur J Biochem.

[b107] Mosyak L, Zhang Y, Glasfeld E, Haney S, Stahl M, Seehra J, Somers WS (2000). The bacterial cell-division protein ZipA and its interaction with an FtsZ fragment revealed by X-ray crystallography. EMBO J.

[b108] Muller T, Rahmann S, Rehmsmeier M (2001). Non-symmetric score matrices and the detection of homologous transmembrane proteins. Bioinformatics.

[b109] Nair R, Rost B (2002a). Inferring sub-cellular localization through automated lexical analysis. Bioinformatics.

[b110] Nair R, Rost B (2002b). Sequence conserved for subcellular localization. Protein Sci.

[b111] Najafabadi HS, Salavati R (2008). Sequence-based prediction of protein–protein interactions by means of codon usage. Genome Biol.

[b112] Nikaido H (2003). Molecular basis of bacterial outer membrane permeability revisited. Microbiol Mol Biol Rev.

[b113] Ou YY, Gromiha MM, Chen SA, Suwa M (2008). TMBETADISC-RBF: discrimination of beta-barrel membrane proteins using RBF networks and PSSM profiles. Comput Biol Chem.

[b114] Pirovano W, Feenstra KA, Heringa J (2008). PRALINETM: a strategy for improved multiple alignment of transmembrane proteins. Bioinformatics.

[b115] Poetsch A, Wolters D (2008). Bacterial membrane proteomics. Proteomics.

[b116] Pratt LA, Kolter R (1998). Genetic analysis of *Escherichia coli* biofilm formation: roles of flagella, motility, chemotaxis and type I pili. Mol Microbiol.

[b117] Pruitt KD, Tatusova T, Maglott DR (2005). NCBI Reference Sequence (RefSeq): a curated non-redundant sequence database of genomes, transcripts and proteins. Nucleic Acids Res.

[b118] Punta M, Forrest LR, Bigelow H, Kernytsky A, Liu J, Rost B (2007). Membrane protein prediction methods. Methods.

[b119] Rajagopala SV, Titz B, Goll J, Parrish JR, Wohlbold K, McKevitt MT, Palzkill T, Mori H, Finley RL, Uetz P (2007). The protein network of bacterial motility. Mol Syst Biol.

[b120] Rangachari K, Davis CT, Eccleston JF, Hirst EM, Saldanha JW, Strath M, Wilson RJ (2002). SufC hydrolyzes ATP and interacts with SufB from *Thermotoga maritima*. FEBS Lett.

[b121] Remaut H, Tang C, Henderson NS, Pinkner JS, Wang T, Hultgren SJ, Thanassi DG, Waksman G, Li H (2008). Fiber formation across the bacterial outer membrane by the chaperone/usher pathway. Cell.

[b122] Rey S, Acab M, Gardy JL, Laird MR, deFays K, Lambert C, Brinkman FS (2005a). PSORTdb: a protein subcellular localization database for bacteria. Nucleic Acids Res.

[b123] Rey S, Gardy JL, Brinkman FS (2005b). Assessing the precision of high-throughput computational and laboratory approaches for the genome-wide identification of protein subcellular localization in bacteria. BMC Genomics.

[b124] Rick PD, Barr K, Sankaran K, Kajimura J, Rush JS, Waechter CJ (2003). Evidence that the wzxE gene of *Escherichia coli* K-12 encodes a protein involved in the transbilayer movement of a trisaccharide-lipid intermediate in the assembly of enterobacterial common antigen. J Biol Chem.

[b125] Rigaut G, Shevchenko A, Rutz B, Wilm M, Mann M, Seraphin B (1999). A generic protein purification method for protein complex characterization and proteome exploration. Nature Biotech.

[b126] Riley M, Abe T, Arnaud MB (2006). *Escherichia coli* K-12: a cooperatively developed annotation snapshot – 2005. Nucleic Acids Res.

[b127] Rizzitello AE, Harper JR, Silhavy TJ (2001). Genetic evidence for parallel pathways of chaperone activity in the periplasm of *Escherichia coli*. J Bacteriol.

[b128] Ruiz N, Gronenberg LS, Kahne D, Silhavy TJ (2008). Identification of two inner-membrane proteins required for the transport of lipopolysaccharide to the outer membrane of *Escherichia coli*. P Natl Acad Sci USA.

[b129] Saier MH (2006). Protein secretion and membrane insertion systems in gram-negative bacteria. J Membr Biol.

[b130] Saier MH, Tran CV, Barabote RD (2006). TCDB: the transporter classification database for membrane transport protein analyses and information. Nucleic Acids Res.

[b131] Salwinski L, Miller CS, Smith AJ, Pettit FK, Bowie JU, Eisenberg D (2004). The database of interacting proteins: 2004 update. Nucleic Acids Res.

[b132] Sansom MS (1999). Membrane proteins: a tale of barrels and corks. Curr Biol.

[b133] Santoni V, Molloy M, Rabilloud T (2000). Membrane proteins and proteomics: un amour impossible?. Electrophoresis.

[b134] Sasarman A, Echelard Y, Letowski J, Tardif D, Drolet M (1988). Nucleotide sequence of the hemX gene, the third member of the Uro operon of *Escherichia coli* K12. Nucleic Acids Res.

[b135] Scheurwater EM, Clarke AJ (2008). The C-terminal domain of *Escherichia coli* YfhD functions as a lytic transglycosylase. J Biol Chem.

[b136] Schleiff E, Soll J (2005). Membrane protein insertion: mixing eukaryotic and prokaryotic concepts. EMBO Rep.

[b137] Schuster SC, Swanson RV, Alex LA, Bourret RB, Simon MI (1993). Assembly and function of a quaternary signal transduction complex monitored by surface plasmon resonance. Nature.

[b138] Schwartz CJ, Djaman O, Imlay JA, Kiley PJ (2000). The cysteine desulfurase, IscS, has a major role in *in vivo* Fe-S cluster formation in Escherichia coli. P Natl Acad Sci USA.

[b139] Schwille P, Bieschke J, Oehlenschlager F (1997). Kinetic investigations by fluorescence correlation spectroscopy: the analytical and diagnostic potential of diffusion studies. Biophys Chem.

[b140] Serres MH, Goswami S, Riley M (2004). GenProtEC: an updated and improved analysis of functions of *Escherichia coli* K-12 proteins. Nucleic Acids Res.

[b141] Shafrir Y, Guy HR (2004). STAM: simple transmembrane alignment method. Bioinformatics.

[b142] Sharan R, Ulitsky I, Shamir R (2007). Network-based prediction of protein function. Mol Syst Biol.

[b143] Shih YL, Rothfield L (2006). The bacterial cytoskeleton. Microbiol Mol Biol Rev.

[b144] Shoemaker BA, Panchenko AR (2007a). Deciphering protein–protein interactions. Part I. Experimental techniques and databases. PLoS Comput Biol.

[b145] Shoemaker BA, Panchenko AR (2007b). Deciphering protein–protein interactions. Part II. Computational methods to predict protein and domain interaction partners. PLoS Comput Biol.

[b146] Sklar JG, Wu T, Kahne D, Silhavy TJ (2007). Defining the roles of the periplasmic chaperones SurA, Skp, and DegP in *Escherichia coli*. Genes Dev.

[b147] Spears KJ, Roe AJ, Gally DL (2006). A comparison of enteropathogenic and enterohaemorrhagic *Escherichia coli* pathogenesis. FEMS Microbiol Lett.

[b148] Spelbrink RE, Kolkman A, Slijper M, Killian JA, de Kruijff B (2005). Detection and identification of stable oligomeric protein complexes in *Escherichia coli* inner membranes: a proteomics approach. J Biol Chem.

[b149] Spory A, Bosserhoff A, von Rhein C, Goebel W, Ludwig A (2002). Differential regulation of multiple proteins of *Escherichia coli* and *Salmonella enterica* serovar Typhimurium by the transcriptional regulator SlyA. J Bacteriol.

[b150] Stenberg F, Chovanec P, Maslen SL, Robinson CV, Ilag LL, von Heijne G, Daley DO (2005). Protein complexes of the *Escherichia coli* cell envelope. J Biol Chem.

[b151] Strauch EM, Georgiou G (2007). A bacterial two-hybrid system based on the twin-arginine transporter pathway of *E. coli*. Protein Sci.

[b152] Sundararaj S, Guo A, Habibi-Nazhad B, Rouani M, Stothard P, Ellison M, Wishart DS (2004). The CyberCell Database (CCDB): a comprehensive, self-updating, relational database to coordinate and facilitate in silico modeling of *Escherichia coli*. Nucleic Acids Res.

[b153] Tamae C, Liu A, Kim K (2008). Determination of antibiotic hypersensitivity among 4,000 single-gene-knockout mutants of *Escherichia coli*. J Bacteriol.

[b154] Taoka M, Yamauchi Y, Shinkawa T, Kaji H, Motohashi W, Nakayama H, Takahashi N, Isobe T (2004). Only a small subset of the horizontally transferred chromosomal genes in *Escherichia coli* are translated into proteins. Mol Cell Proteomics.

[b155] Tatusov RL, Galperin MY, Natale DA, Koonin EV (2000). The COG database: a tool for genome-scale analysis of protein functions and evolution. Nucleic Acids Res.

[b156] Thanassi DG, Stathopoulos C, Karkal A, Li H (2005). Protein secretion in the absence of ATP: the autotransporter, two-partner secretion and chaperone/usher pathways of gram-negative bacteria (review). Mol Membr Biol.

[b157] Thompson A, Schafer J, Kuhn K, Kienle S, Schwarz J, Schmidt G, Neumann T, Johnstone R, Mohammed AK, Hamon C (2003). Tandem mass tags: a novel quantification strategy for comparative analysis of complex protein mixtures by MS/MS. Anal Chem.

[b158] Tikhonova EB, Zgurskaya HI (2004). AcrA, AcrB, and TolC of *Escherichia coli* form a stable intermembrane multidrug efflux complex. J Biol Chem.

[b159] Tokuda H, Matsuyama S (2004). Sorting of lipoproteins to the outer membrane in *E. coli*. Biochim Biophys Acta.

[b160] Tusnady GE, Simon I (1998). Principles governing amino acid composition of integral membrane proteins: application to topology prediction. J Mol Biol.

[b161] Tusnády GE, Kalmár L, Simon I (2008). TOPDB: topology data bank of transmembrane proteins. Nucleic Acids Res.

[b162] Typas A, Nichols RJ, Siegele DA (2008). High-throughput, quantitative analyses of genetic interactions in *E. coli*. Nat Methods.

[b163] Vaknin A, Berg HC (2004). Single-cell FRET imaging of phosphatase activity in the *Escherichia coli* chemotaxis system. P Natl Acad Sci USA.

[b164] van der Laan M, Houben EN, Nouwen N, Luirink J, Driessen AJ (2001). Reconstitution of Sec-dependent membrane protein insertion: nascent FtsQ interacts with YidC in a SecYEG-dependent manner. EMBO Rep.

[b165] Veldhuis G, Hink M, Krasnikov V, van den Bogaart G, Hoeboer J, Visser AJ, Broos J, Poolman B (2006). The oligomeric state and stability of the mannitol transporter, EnzymeII(mtl), from *Escherichia coli*: a fluorescence correlation spectroscopy study. Protein Sci.

[b166] Visudtiphole V, Chalton DA, Hong Q, Lakey JH (2006). Determining OMP topology by computation, surface plasmon resonance and cysteine labelling: the test case of OMPG. Biochem Biophys Res Commun.

[b167] Volker U, Hecker M (2005). From genomics via proteomics to cellular physiology of the gram-positive model organism *Bacillus subtilis*. Cell Microbiol.

[b168] von Mering C, Jensen LJ, Kuhn M, Chaffron S, Doerks T, Kruger B, Snel B, Bork P (2007). STRING 7 – recent developments in the integration and prediction of protein interactions. Nucleic Acids Res.

[b169] Wagner K, Racaityte K, Unger KK, Miliotis T, Edholm LE, Bischoff R, Marko-Varga G (2000). Protein mapping by two-dimensional high performance liquid chromatography. J Chromatogr A.

[b170] Wallin E, von Heijne G (1998). Genome-wide analysis of integral membrane proteins from eubacterial, archaean, and eukaryotic organisms. Protein Sci.

[b171] Wang J, Sung WK, Krishnan A, Li KB (2005). Protein subcellular localization prediction for gram-negative bacteria using amino acid subalphabets and a combination of multiple support vector machines. BMC Bioinformatics.

[b172] Weiner JH, Li L (2008). Proteome of the *Escherichia coli* envelope and technological challenges in membrane proteome analysis. Biochim Biophys Acta.

[b173] Werner J, Misra R (2005). YaeT (Omp85) affects the assembly of lipid-dependent and lipid-independent outer membrane proteins of *Escherichia coli*. Mol Microbiol.

[b174] Wimley WC (2003). The versatile beta-barrel membrane protein. Curr Opin Struct Biol.

[b175] Wu CC, MacCoss MJ, Howell KE, Yates JR (2003). A method for the comprehensive proteomic analysis of membrane proteins. Nat Biotechnol.

[b176] Wu T, Malinverni J, Ruiz N, Kim S, Silhavy TJ, Kahne D (2005). Identification of a multicomponent complex required for outer membrane biogenesis in *Escherichia coli*. Cell.

[b177] Xenarios I, Rice DW, Salwinski L, Baron MK, Marcotte EM, Eisenberg D (2000). DIP: the database of interacting proteins. Nucleic Acids Res.

[b178] Yan A, Guan Z, Raetz CR (2007). An undecaprenyl phosphate-aminoarabinose flippase required for polymyxin resistance in *Escherichia coli*. J Biol Chem.

[b179] Yan JX, Devenish AT, Wait R, Stone T, Lewis S, Fowler S (2002). Fluorescence two-dimensional difference gel electrophoresis and mass spectrometry based proteomic analysis of *Escherichia coli*. Proteomics.

[b180] Yang J, Chen L, Sun L, Yu J, Jin Q (2008). VFDB 2008 release: an enhanced web-based resource for comparative pathogenomics. Nucleic Acids Res.

[b181] Yellaboina S, Goyal K, Mande SC (2007). Inferring genome-wide functional linkages in *E. coli* by combining improved genome context methods: comparison with high-throughput experimental data. Genome Res.

[b182] Yokota N, Kuroda T, Matsuyama S, Tokuda H (1999). Characterization of the LolA–LolB system as the general lipoprotein localization mechanism of *Escherichia coli*. J Biol Chem.

[b183] Yu CS, Lin CJ, Hwang JK (2004). Predicting subcellular localization of proteins for gram-negative bacteria by support vector machines based on *n*-peptide compositions. Protein Sci.

[b184] Yu CS, Chen YC, Lu CH, Hwang JK (2006). Prediction of protein subcellular localization. Proteins.

[b185] Zeghouf M, Li J, Butland G, Borskowska A, Canadien V, Richards D, Beattie B, Emili A, Greenblatt JF (2004). Sequential peptide affinity (SPA) system for the identification of mammalian and bacterial protein complexes. J Proteome Res.

[b186] Zhang N, Chen R, Young N, Wishart D, Winter P, Weiner JH, Li L (2007). Comparison of SDS- and methanol-assisted protein solubilization and digestion methods for *Escherichia coli* membrane proteome analysis by 2-D LC-MS/MS. Proteomics.

[b187] Zhang Z, Henzel WJ (2004). Signal peptide prediction based on analysis of experimentally verified cleavage sites. Protein Sci.

[b188] Zhou M, Boekhorst J, Francke C, Siezen RJ (2008). LocateP: genome-scale subcellular-location predictor for bacterial proteins. BMC Bioinformatics.

